# Low-Dose LPS Induces Tolerogenic Treg Skewing in Asthma

**DOI:** 10.3389/fimmu.2020.02150

**Published:** 2020-09-23

**Authors:** Fengxia Ding, Bo Liu, Chao Niu, Ting Wang, Yaping Wang, Gang Geng, Daiyin Tian, Jihong Dai, Zhou Fu

**Affiliations:** ^1^Department of Pediatric Respiratory Medicine, Children’s Hospital of Chongqing Medical University, Chongqing, China; ^2^Ministry of Education Key Laboratory of Child Development and Disorders, Chongqing, China; ^3^China International Science and Technology Cooperation Base of Child Development and Critical Disorders, Chongqing, China; ^4^Chongqing Key Laboratory of Child Infection and Immunity, Chongqing, China; ^5^Department of Cardiothoracic Surgery, Children’s Hospital of Chongqing Medical University, Chongqing, China

**Keywords:** asthma, LPS, endotoxin tolerance, TLR4, GITRL

## Abstract

The mechanism(s) underlying endotoxin tolerance in asthma remain elusive. As the endotoxin lipopolysaccharide (LPS) affects the expression of the regulatory T-cell (Treg)-suppressive glucocorticoid-induced tumor necrosis factor receptor ligand (GITRL) on antigen-presenting dendritic cells (DCs), we hypothesized that LPS-induced changes in DC GITRL expression may impact Treg-mediated T-helper (Th) cell suppression and the induction of endotoxin tolerance. Here, we propose a novel mechanism by which low-dose LPS inhalation in neonatal mice induces endotoxin tolerance, thereby offering protection from later asthma development. Three-day old wild-type and Toll-like receptor 4 (TLR4)-deficient neonatal mice were exposed to low-dose LPS (1 μg) intranasally for 10 consecutive days prior to ovalbumin (OVA)-induced asthma to better understand the tolerogenic mechanism(s) of low-dose LPS pre-exposure. *In vivo* findings were validated using *in vitro* co-culturing studies of primary CD11c^+^ DCs and CD4^+^ T-cells with or without low-dose LPS pre-exposure before OVA stimulation. Low-dose LPS pre-exposure upregulated the Treg response and downregulated pathogenic Th2 and Th17 responses through promoting apoptosis of Th2 and Th17 cells. Low-dose LPS pre-exposure downregulated DC GITRL expression and T-cell GITR expression. Artificial DC GITRL expression abrogated the tolerogenic Treg-skewing effect of low-dose LPS pre-exposure. Low-dose LPS pre-exposure inhibited TRIF/IRF3/IFNβ signaling and upregulated expression of tolerogenic TRIF/IRF3/IFNβ negative regulators in a TLR4-dependent manner. This tolerogenic DC GITRL downregulation was attributable to TRIF/IRF3/IFNβ signaling inhibition. Low-dose LPS pre-exposure produces tolerogenic Treg skewing in neonatal asthmatic mice, a phenomenon attributable to TLR4-dependent TRIF/IRF3/IFNβ-mediated DC GITRL downregulation.

## Introduction

Asthma is an allergen-derived immunologic disorder characterized by airway hyperresponsiveness (AHR), chronic airway inflammation, and enhanced allergen-specific IgE production (1). Although the incidence of asthma has been increasing globally (2), asthma medications are mainly anti-symptomatic, and allergen-specific immunotherapy does not show strong evidence of efficacy (3). Thus, blocking the effects of the immune response in the critical period before asthma formation has become an important issue for both researchers and clinicians.

Notably, epidemiological studies have demonstrated that children exposed to environments rich in the endotoxin lipopolysaccharide (LPS) have a lower prevalence of asthma and other allergic diseases, a phenomenon termed endotoxin tolerance (4, 5). For instance, environmental endotoxin exposure, such as from LPS present in dust from mattresses or kitchen floors, has been shown to protect against asthma and atopy in children residing in farming and non-farming households (4, 6). On this basis, LPS may be a valuable tool in inducing endotoxin tolerance prior to asthma development. However, a recent study has demonstrated that differing levels of LPS exposure can produce divergent immune responses in murine models of asthma (7). Therefore, the precise mechanism(s) underlying endotoxin tolerance remain unclear and require elucidation.

CD4^+^ T-cells, comprised mainly of T-helper (Th)1, Th2, Th17, and regulatory T (Treg) cells, are critical cellular mediators of asthma (8). Although an impaired Th1/Th2 balance in favor of Th2 cells has been clearly established in asthma patients (9), the role of Treg cells has also gained interest among asthma researchers (10, 11). As Treg cells function in opposition to T-helper cells, their main function in asthma is to negatively regulate Th1, Th2, and Th17 cells to prevent Th-cell hyperactivity (10, 11). Accordingly, targeted depletion of Treg cells has been shown to aggravate a murine model of asthma (12), while adoptive transfer of induced Treg cells produces tolerogenic effects (13, 14). As early childhood exposure to LPS-expressing microorganisms induces Treg cells and suppresses aberrant Th2 immune responses (15), Treg-mediated Th cell suppression may play a critical role in the induction of endotoxin tolerance. However, the molecular mechanism(s) underlying this tolerogenic phenomenon remain unclear.

Dendritic cells (DCs) are allergen-sensing, antigen-presenting cells (APCs) that activate T-cells and direct their differentiation toward Th1, Th2, Th17, or Treg lineages (15). One key co-stimulatory molecule present on the surface of DCs—glucocorticoid-induced tumor necrosis factor receptor ligand (GITRL)—*via* binding to its corresponding T-cell ligand GITR serves to inhibit Treg-mediated Th cell suppression and enhance Th2 cell activity, thus augmenting AHR, serum IgE levels, and Th2 cytokine release in a murine model of asthma (16). As LPS has been shown to affect GITRL expression on DCs (17), we hypothesized that LPS-induced changes in DC GITRL expression may impact Treg-mediated Th cell suppression and the induction of endotoxin tolerance. In this study, we propose a novel mechanism by which low-dose LPS inhalation in neonatal mice induces endotoxin tolerance, thereby offering protection from later asthma development.

## Materials and Methods

The methods are fully detailed in the Supplementary Material.

## Results

### Construction of Optimal LPS Pre-exposure Model

**FIGURE 1:**
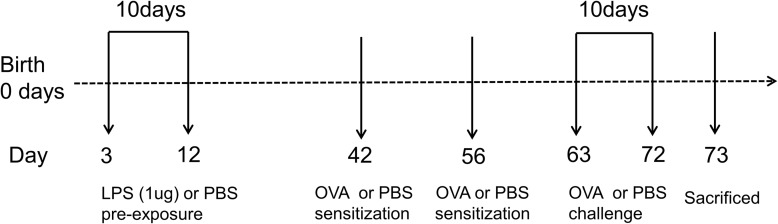
Experimental protocol for constructing murine model of asthma. Neonatal BALB/c mice received daily low-dose LPS (1 μg) or PBS (control) intranasally (i.n.) from the 3rd DOL for a period of 10 consecutive days. Mice were then sensitized with 100 μg OVA plus 100 μl aluminum hydroxide (AlOH) or PBS (control) *via* intraperitoneal (i.p.) injection on the 42nd DOL and 56th DOL, and then further exposed to a daily, 30-min 1% OVA aerosol or PBS aerosol (control) from the 63rd DOL for a period of 10 consecutive days. Twenty-four hours after the last OVA challenge, the mice were sacrificed. *n* = 6–8 mice per group.

To explore the effect of LPS pre-exposure during immune maturation in early life, neonatal mice were pre-exposed to two different doses of LPS (1 or 100 μg) at two different time points after birth 3rd or 14th day of life (DOL) before the ovalbumin (OVA)-induced asthma model was established (Supplementary Figure 1). These various LPS pre-exposure protocols exhibited different effects on asthma development. Notably, newborn mice with the lower dose of LPS (3d1μgLPS/OVA) were significantly protected from asthma with significantly reduced AHR (*p* < 0.05, Supplementary Figure 2A), significantly reduced peribronchial and perivascular inflammation in lung tissues (*p* < 0.05, Supplementary Figure 2B), significantly reduced serum OVA-specific IgE levels (*p* < 0.05, Supplementary Figure 2C), and significantly reduced bronchoalveolar lavage fluid (BALF) levels of pro-eosinophilic/neutrophilic cytokines (*p* < 0.05, Supplementary Figure 3C). Thus, the 3d1μgLPS/OVA model was chosen as the optimal LPS pre-exposure model for subsequent experiments (Figure 1).

### LPS Pre-exposure Promotes Treg Skewing *in vivo*

Although the exact mechanism(s) underlying endotoxin tolerance in asthma are still unclear, current evidence suggests that T-cell distributions favoring Treg (CD4^+^CD25^+^Foxp3^+^) or Th1 cells (which are induced by endotoxin exposure) over pathogenic Th2 (CD4^+^IL-4^+^) and Th17 (CD4^+^IL-17^+^) cells, as well as the suppression of DCs and barrier epithelial cells, may play important roles in the development of endotoxin tolerance (18). Therefore, using the aforementioned 3d1μgLPS/OVA model, we assessed the changes of CD4^+^ T-cell profiles between control, PBS/OVA, 3d1μgLPS/OVA, and 3d1μgLPS/PBS mice. After asthma induction, the proportion of Treg cells and Treg-associated Foxp3 mRNA expression significantly decreased, whereas the proportions of Th2 cells (and Th2-associated GATA3 mRNA expression) as well as Th17 cells (and Th17-associated ROR-γt mRNA expression) significantly increased in PBS/OVA mice relative to the control group (*p* < 0.05, Supplementary Figures 3A–B). Accordingly, asthma induction also significantly decreased Treg-associated IL-10 and TGF-β production while significantly increasing Th2-associated IL-4, IL-5, and IL-13 production and Th17-associated IL-17 production (*p* < 0.05, Supplementary Figure 3C). Consistent with our hypothesis, the 3d1μgLPS/OVA mice displayed significant reversal of these asthma-induced effects, with significant increases in the proportion of Treg cells (and their associated markers) and significant decreases in the proportions of Th2 and Th17 cells (and their associated markers) as compared to those of PBS/OVA mice (*p* < 0.05, Supplementary Figures 3A–C). However, simple exposure to 1 μg LPS without asthma induction (LPS/PBS) did not significantly affect Foxp3, GATA3, or ROR-γt mRNA levels nor IL-10, TGF-β, IL-4, IL-5, IL-13, or IL-17 levels (*p* > 0.05, Supplementary Figures 3A–C) compared to those of Control mice. These results demonstrate that pre-exposure with low-dose LPS upregulates the Treg response and downregulates Th2 and Th17 responses, thereby producing positive Treg skewing in neonatal asthmatic mice.

### LPS Pre-exposure Increases Apoptosis of Th2 and Th17 Cells *in vitro*

Having shown that low-dose LPS pre-exposure produces Treg skewing *in vivo*, we next examined the effects of low-dose LPS pre-exposure on apoptosis levels of the CD4^+^ T-cell subsets *in vitro*. We separated Treg, Th1, Th2, and Th17 cells by flow cytometry and respectively analyzed cleaved caspase-3 expression and apoptotic cell percentages in these subsets (Supplementary Figure 4). Cleaved caspase-3 expression and apoptotic cell percentages of Th2 and Th17 cells were significantly decreased under PBS/OVA conditions as compared to control conditions (*p* < 0.05, Supplementary Figures 4C,D). Notably, cleaved caspase-3 expression and apoptotic cell percentages of Th2 and Th17 cells increased significantly in LPS/OVA cells as compared with PBS/OVA cells (*p* < 0.05, Supplementary Figures 4C,D). However, no significant changes in cleaved caspase-3 expression or apoptotic cell percentages were observed in Treg cells or Th1 cells (*p* > 0.05, Supplementary Figures 4A,B). These results support our *in vivo* findings that pre-exposure with low-dose LPS exposure produces Treg skewing, likely through promoting apoptosis of pathogenic Th2 and Th17 cells.

### LPS Pre-exposure Downregulates DC GITRL and T-Cell GITR Expression *in vivo*

**FIGURE 2:**
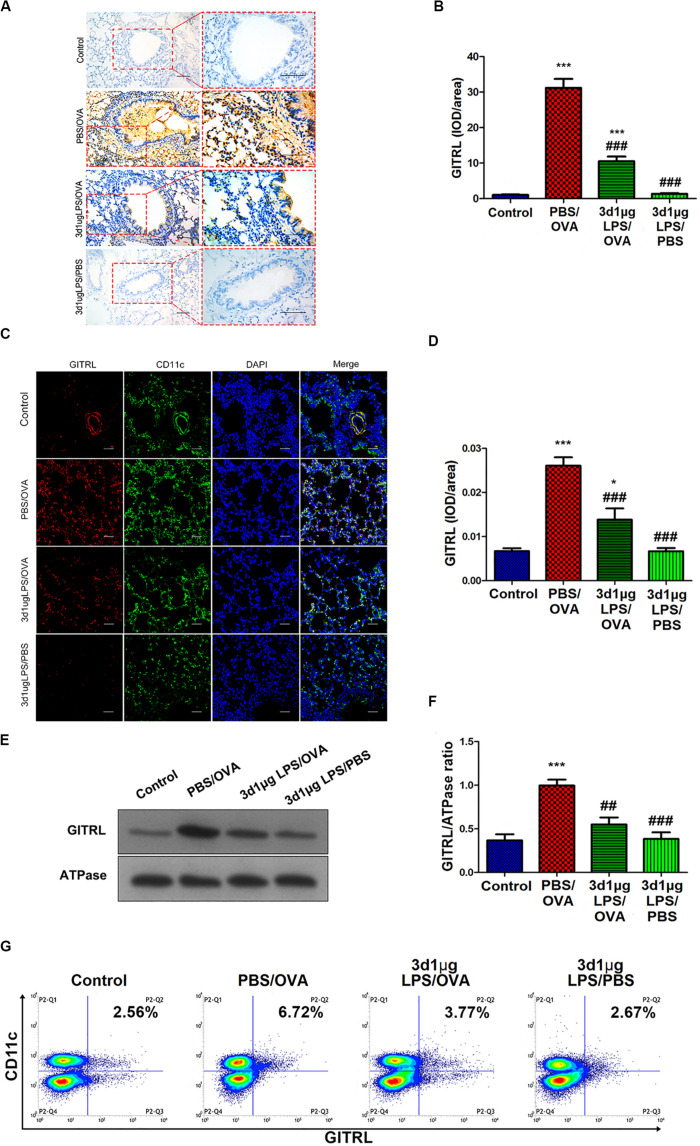
Lung-derived dendritic cell GITRL expression downregulated in low-dose LPS-pre-exposed asthmatic mice relative to untreated asthmatic mice. **(A,B)** Immunohistochemistry of GITRL in lung tissue sections. Left panel (200× magnification) scale bars = 50 μm, right panel (400× magnification) scale bars = 100 μm. **(C,D)** Immunofluorescence of GITRL in lung tissue sections. Scale bars (200× magnification) = 50 μm. **(E,F)** Membrane-fraction immunoblotting of GITRL expression in lung-derived CD11c^+^CD11b^+^ dendritic cells. **(G)** Flow cytometry of surface GITRL expression on lung-derived CD11c^+^CD11b^+^ dendritic cells. *n* = 6–8 mice per group. Data are reported as means ± standard deviations (SDs). *p < 0.05 and ***p < 0.001 vs. Control group; ^##^*p* < 0.01 and ^###^*p* < 0.001 vs. PBS/OVA group. Control, unexposed normal mice; PBS/OVA, asthmatic mice; 3d1μgLPS/OVA, low-dose (1 μg daily) LPS-exposed asthmatic mice with LPS exposure at 3rd DOL; 3d1μgLPS/PBS, low-dose (1 μg daily) LPS-exposed normal mice with LPS exposure at 3rd DOL.

Stimulation of Treg-expressed GITR by DC-expressed GITRL has been shown to abolish Treg suppression and increase the proliferation of effector T-cells resistant to Treg suppressive activity (16), and LPS has been shown to affect GITRL expression on DCs (17). We hypothesized that the rebalancing between Treg cells and effector T-cells observed after pre-exposure with low-dose LPS in asthmatic mice may be due to altered GITR and GITRL expression. Indeed, we found that GITRL expression in lung-derived DCs from 3d1μgLPS/OVA mice was significantly lower than that in PBS control mice according to immunohistochemistry (*p* < 0.05, Figures 2A,B), immunofluorescence (*p* < 0.05, Figures 2C,D), membrane-fraction western blotting (*p* < 0.05, Figures 2E,F), and flow cytometry (2.53 ± 0.46 vs. 6.82 ± 0.78 vs. 3.43 ± 0.74 vs. 2.55 ± 0.63, *p* < 0.05, Figure 2G).

We further studied GITR expression on Tregs as well as Th1, Th2, and Th17 cells extracted from mice. Mirroring GITRL expression on DCs, our results show that the levels of GITR on all T-cell subtypes tested in 3d1μgLPS/OVA mice were significantly lower than those in PBS/OVA mice (*p* < 0.05, Supplementary Figure 5). These results show that DC GITRL expression and T-cell GITR expression are downregulated *in vivo* by pre-exposure with low-dose LPS in our murine model of asthma.

### LPS Pre-exposure Downregulates DC GITRL and T-Cell GITR Expression *in vitro*

**FIGURE 3:**
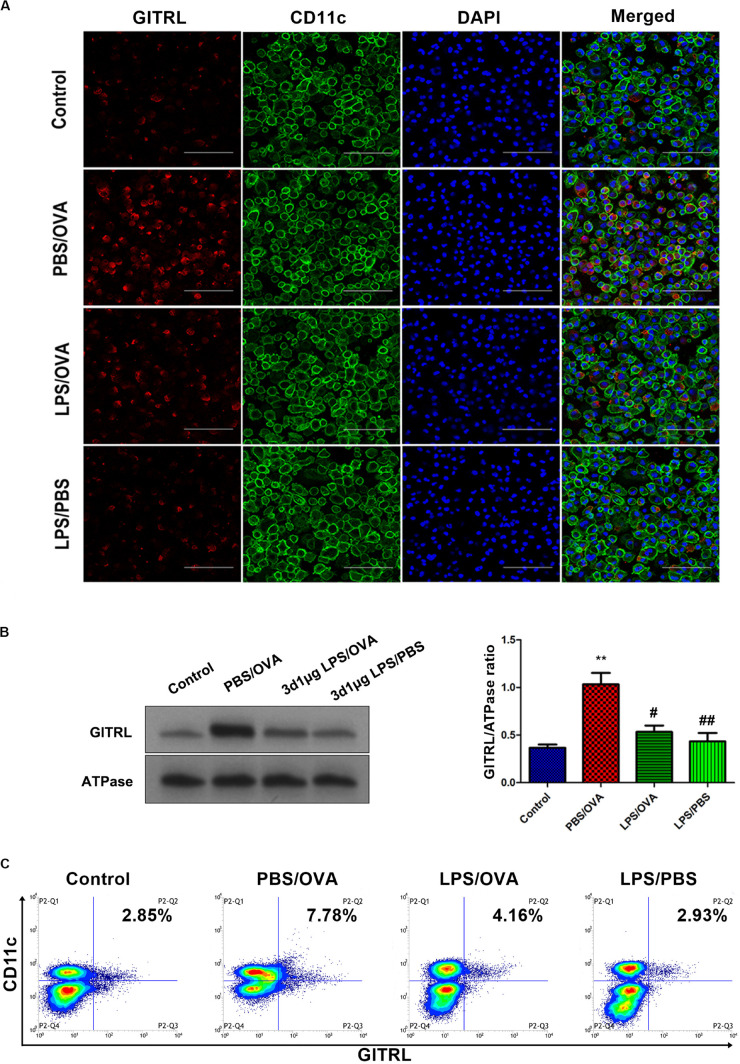
Co-culture of low-dose LPS-pre-exposed dendritic cells and T-cells prior to OVA stimulation downregulates dendritic cell GITRL expression. GITRL expression on CD11c^+^CD11b^+^ dendritic cells were significantly downregulated in the low-dose LPS-exposed LPS/OVA cells (100 ng/ml LPS) as compared with PBS/OVA cells. **(A)** Surface GITRL expression on primary dendritic cells by immunofluorescence. Scale bars (600× magnification) = 150 μm. **(B)** Membrane-fraction immunoblotting of GITRL expression on primary dendritic cells. **(C)** Surface GITRL expression on primary dendritic cells by flow cytometry. Data are reported as means ± standard deviations (SDs). **p < 0.01 vs. Control group; ^#^*p* < 0.05 and ^##^*p* < 0.01 vs. PBS/OVA group. Control, unexposed normal mice; PBS/OVA, asthmatic mice; LPS/OVA, low-dose LPS-exposed asthmatic mice; LPS/PBS, low-dose LPS-exposed normal mice.

In order to validate our *in vivo* findings, we further confirmed the effects of low-dose LPS pre-exposure on DC GITRL expression and T-cell GITR expression using *in vitro* co-culturing studies. Primary CD11c^+^CD11b^+^ DCs and CD4^+^ T-cells were extracted and sorted for co-culture with or without low-dose LPS pre-exposure (100 ng/ml) before OVA peptide stimulation (1 μg/ml). Employing immunofluorescence, membrane-fraction Western blotting, and flow cytometry, we demonstrated that GITRL expression on PBS/OVA DCs was higher than control DCs (*p* < 0.05, Figures 3A–C), and expression of GITRL significantly decreased after pre-exposure with low-dose LPS (LPS/OVA) (*p* < 0.05, Figures 3A–C). In parallel, the levels of GITR on Treg, Th2, and Th17 cells in PBS/OVA mice were significantly higher than matching cells from control mice, whereas the GITR expression was significantly decreased after pre-exposure with low-dose LPS (LPS/OVA) (Supplementary Figure 6, *p* < 0.05). Notably, GITR expression on Th1 cells was not significantly different between the experimental groups (Supplementary Figure 6B, *p* > 0.05). These *in vitro* results confirm that DC GITRL expression and T-cell GITR expression are downregulated by pre-exposure to low-dose LPS.

### Artificial Overexpression of DC GITRL Abrogates the Tolerogenic Treg-Skewing Effect of Low-Dose LPS Pre-exposure

**FIGURE 4:**
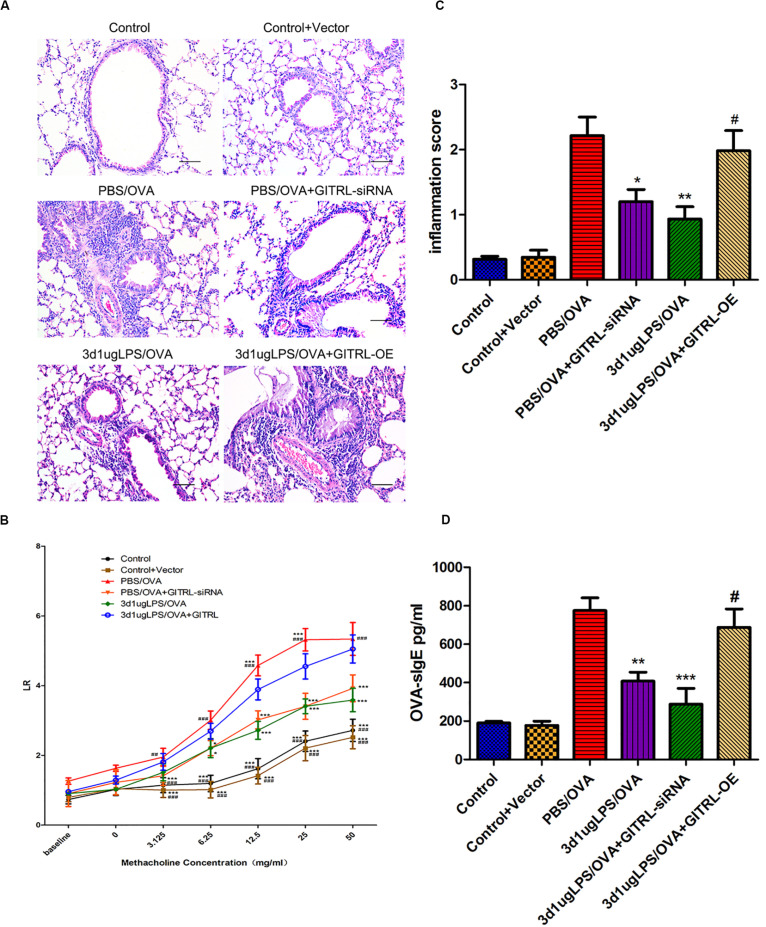
Pulmonary effects of GITRL silencing or overexpression on low-dose LPS-pre-exposed asthmatic mice. **(A)** Representative images of lung tissue sections stained with hematoxylin and eosin (H&E) 24 h after the final challenge. Left panel (200× magnification) scale bars = 50 μm, right panel (400× magnification) scale bars = 100 μm. **(B)** Lung resistance (LR) values in reaction to increasing doses of methacholine were measured 24 h after the final challenge. **(C)** Inflammation score in each group. **(D)** Serum OVA-specific IgE levels as measured by ELISA. *n* = 6–8 mice per group. Data are reported as means ± standard deviations (SDs). *p < 0.05, **p < 0.01, and ***p < 0.001 vs. PBS/OVA group; ^#^*p* < 0.05, ^##^*p* < 0.01, and ^###^*p* < 0.001 vs. 3d1μgLPS/OVA group. Control, unexposed normal mice; Control + Vector, unexposed normal mice with empty vector; PBS/OVA, asthmatic mice; 3d1μgLPS/OVA, low-dose LPS-exposed asthmatic mice; 3d1μgLPS/OVA + GITRL-siRNA, low dose LPS-exposed asthmatic mice with GITRL-siRNA DCs; 3d1μgLPS/OVA + GITRL-OE, low dose LPS-exposed asthmatic mice with GITRL-overexpressing DCs.

To determine whether the effects of low-dose LPS pre-exposure are GITRL-dependent, we next altered bone-marrow-derived DC GITRL expression by transfection with either a GITRL siRNA or a recombinant pEGFP-N1-GITRL overexpression plasmid and then adoptively transferred these transfected DCs into mice. To validate that the DCs successfully transferred and migrated to the lung, we confirmed stable GITRL transcript knockdown and overexpression in lung-derived DCs from GITRL-silenced 3d1μgLPS/OVA and GITRL-overexpressing 3d1μgLPS/OVA mice, respectively (*p* < 0.05, Supplementary Figure 7).

glucocorticoid-induced tumor necrosis factor receptor ligand-overexpressing 3d1μgLPS/OVA mice showed significantly higher peribronchial and perivascular inflammation in lung tissues (*p* < 0.05, Figure 4A), significantly higher levels of AHR (*p* < 0.05, Figure 4B), significantly higher inflammation score (*p* < 0.05, Figure 4C), significantly higher serum OVA-specific IgE levels (*p* < 0.05, Figure 4D) and significantly higher BALF levels of pro-eosinophilic/neutrophilic cytokines (*p* < 0.05, Supplementary Figure 8C) as compared to 3d1μgLPS/OVA mice. Moreover, GITRL-overexpressing 3d1μgLPS/OVA mice displayed significant decreases in Treg levels and Treg-associated Foxp3 mRNA expression accompanied by significant increases in Th2 levels and Th2-associated GATA3 mRNA expression as well as significant increases in Th17 levels and Th17-associated ROR-γt mRNA expression as compared to 3d1μgLPS/OVA mice (*p* < 0.05, Supplementary Figures 8A,B). Changes in BALF cytokine levels paralleled the changes in T-cell subset composition (*p* < 0.05, Supplementary Figure 8C). The opposite effects were observed in GITRL-silenced 3d1μgLPS/OVA mice (*p* < 0.05, Figures 4A–C and Supplementary Figures 8A–C).

In order to validate our *in vivo* findings, we further confirmed the effects of altered GITRL expression using *in vitro* co-culturing studies. Primary transfected CD11c^+^CD11b^+^ DCs and CD4^+^ T-cells were co-cultured with or without low-dose LPS pre-exposure before OVA stimulation. We first validated stable GITRL transcript knockdown and overexpression in these primary transfected DCs (*p* < 0.05, Supplementary Figure 9). Similar to our *in vivo* findings, GITRL-overexpressing LPS/OVA DCs produced significant decreases in Treg levels and Treg-associated Foxp3 mRNA expression accompanied by significant increases in Th2 levels and Th2-associated GATA3 mRNA expression as well as significant increases in Th17 levels and Th17-associated ROR-γt mRNA expression as compared to LPS/OVA DCs (*p* < 0.05, Supplementary Figures 10A,B). Changes in BALF cytokine levels paralleled the changes in T-cell subset composition (*p* < 0.05, Supplementary Figure 10C). The opposite effects were observed with GITRL-silenced LPS/OVA DCs (*p* < 0.05, Supplementary Figures 10A–C). These combined results demonstrate that the tolerogenic Treg-skewing effect of low-dose LPS pre-exposure is abrogated by artificial DC GITRL expression.

### LPS Pre-exposure Downregulates DC TRIF/IRF3/IFNβ Pathway Activation in a TLR4-Dependent Manner

Having shown that the tolerogenic effects of low-dose LPS pre-exposure are due to GITRL downregulation on DCs, we next examined the molecular mechanism(s) underlying DC GITRL downregulation following low-dose LPS pre-exposure. LPS binds to Toll-like receptor 4 (TLR4) expressed on the surface of APCs, and this LPS-TLR4 ligation regulates the downstream TRIF/IRF3/IFNβ (TIR-domain-containing adapter-inducing interferon-β/interferon regulatory factor 3/interferon-β) pathway *via* multiple negative feedback loops (19, 20). Given the fact that IFNβ stimulates GITRL expression (21), we hypothesized that TLR4-dependent TRIF/IRF3/IFNβ signaling inhibition is responsible for the GITRL downregulation observed in low-dose LPS pre-exposed DCs.

**FIGURE 5:**
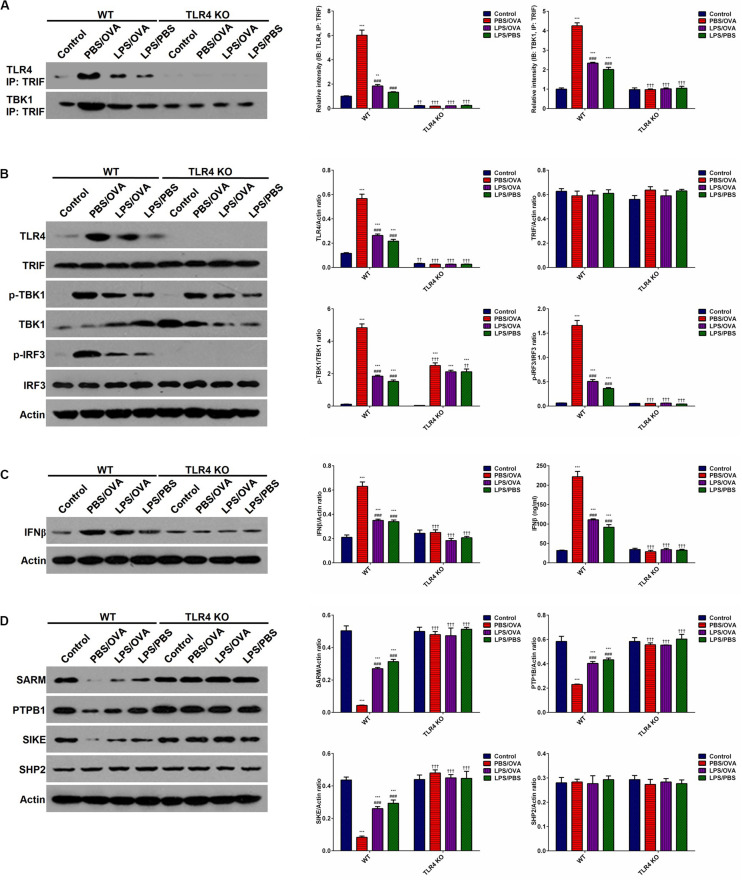
Low-dose LPS pre-exposure downregulates TLR4-dependent TRIF/IRF3/IFNβ pathway activation in dendritic cells. TLR4-mediated TRIF/IRF3/IFNβ pathway activation in CD11c^+^CD11b^+^ dendritic cells was significantly downregulated in the low-dose LPS-exposed LPS/OVA cells (100 ng/ml LPS) as compared with PBS/OVA cells. **(A)** Whole-cell lysate immunoblotting of TLR4 and TBK1 following immunoprecipitation with anti-TRIF antibodies in primary dendritic cells. **(B)** Whole-cell lysate immunoblotting of TLR4/TRIF/TBK1/IRF3 pathway proteins in primary dendritic cells. **(C)** Whole-cell lysate immunoblotting of IFNβ (left panels) and supernatant levels of IFNβ as measured by ELISA (right panel) in primary dendritic cells. **(D)** Whole-cell lysate immunoblotting of four key negative regulators of the TLR4/TRIF/TBK1/IRF3 pathway proteins in primary dendritic cells. Data are reported as means ± standard deviations (SDs). **p < 0.01 and ***p < 0.001 vs. Control group; ^###^*p* < 0.001 vs. PBS/OVA group; ^††^*p* < 0.01 and ^†††^*p* < 0.001 vs. WT.

To determine whether the effects of low-dose LPS pre-exposure are TLR4-dependent, we next obtained TLR4-deficient CD11c^+^CD11b^+^ DCs from the bone marrow of TLR4 knockout (KO) mice. Then, either wild-type (WT) DCs or TLR4-KO DCs were co-cultured with CD4^+^ T-cells with or without low-dose LPS pre-exposure before OVA stimulation in order to determine the downstream effects of TLR4. We first validated stable TLR4 expression and stable TLR4 knockdown in the WT and TLR4-KO DCs, respectively, *via* immunoprecipitation with TRIF and simple immunoblotting (*p* < 0.05, Figures 5A,B). PBS/OVA WT DCs showed significantly higher activation of the downstream TRIF/IRF3/IFNβ signaling cascade compared to control DCs (*p* < 0.05, Figures 5B,C). Notably, TRIF/IRF3/IFNβ signaling was significantly decreased in WT DCs after pre-exposure with low-dose LPS (LPS/OVA) (*p* < 0.05, Figures 5B,C). In contrast, this inhibitory effect of low-dose LPS pre-exposure on TRIF/IRF3/IFNβ signaling was completely abrogated in TLR4-KO DCs (*p* > 0.05, Figures 5B,C). These results demonstrate that the inhibitory effect of low-dose LPS pre-exposure on downstream TRIF/IRF3/IFNβ signaling in DCs is TLR4-dependent.

In order to further investigate the mechanism(s) by which low-dose LPS pre-exposure affects TRIF/IRF3/IFNβ signaling in DCs, we next measured the expression of several TLR4-associated negative regulators of TRIF/IRF3/IFNβ signaling, including SARM (Sterile alpha and armadillo-motif containing protein), PTPB1 (Polypyrimidine-tract binding protein), SIKE (Suppressor of IKK epsilon), and SHP2 (Src homology region 2-containing protein tyrosine phosphatase 2) (22–24). PBS/OVA WT DCs showed significant downregulation of SARM, PTPB1, and SIKE compared to control DCs (*p* < 0.05, Figure 5D). Notably, LPS/OVA WT DCs displayed significant upregulation of these negative regulators compared to PBS/OVA WT DCs (*p* < 0.05, Figure 5D). In contrast, this upregulating effect of low-dose LPS pre-exposure on SARM, PTPB1, and SIKE expression was completely abrogated in TLR4-KO DCs (*p* > 0.05, Figure 5D). These results suggest that the inhibitory effect of low-dose LPS pre-exposure on TRIF/IRF3/IFNβ signaling in DCs may be mediated by the negative regulators SARM, PTPB1, and SIKE in a TLR4-dependent manner.

In order to further validate our findings, we took a deeper look at IRF3. It is well-established that TBK1-phosphorylated IRF3 dimerizes in the cytoplasm and translocates to the nucleus, where it transactivates several potent genes, most notably IFNβ (22). Here, we validated that PBS/OVA WT DCs displayed significantly higher IRF3 dimerization, nuclear translocation, and transactivation potential compared to control DCs (*p* < 0.05, Supplementary Figures 11A–C). Notably, these effects were significantly decreased in WT DCs after pre-exposure with low-dose LPS (LPS/OVA) (*p* < 0.05, Supplementary Figures 11A–C). In contrast, these inhibitory effects of low-dose LPS pre-exposure on IRF3 activation were completely abrogated in TLR4-KO DCs (*p* > 0.05, Supplementary Figures 11A–C).

### IFNβ Exposure Rescues Dendritic Cell GITRL Downregulation From Low-Dose LPS Pre-exposure

**FIGURE 6:**
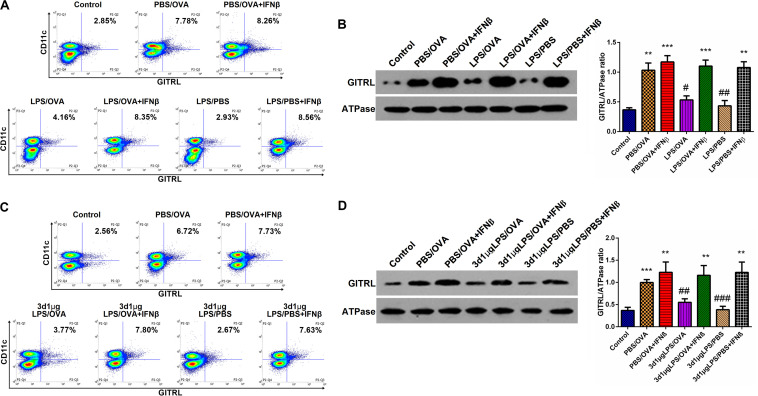
IFNβ exposure rescues dendritic cell GITRL downregulation from low-dose LPS pre-exposure. **(A)** Surface GITRL expression on primary CD11c^+^CD11b^+^ dendritic cells by flow cytometry. **(B)** Membrane-fraction immunoblotting of GITRL expression on primary CD11c^+^CD11b^+^ dendritic cells. **(C)** Flow cytometry of surface GITRL expression on lung-derived CD11c^+^CD11b^+^ dendritic cells. **(D)** Membrane-fraction immunoblotting of GITRL expression in lung-derived CD11c^+^CD11b^+^ dendritic cells. Data are reported as means ± standard deviations (SDs). **p < 0.01 and ***p < 0.001 vs. Control group; ^#^*p* < 0.05, ^##^*p* < 0.01, and ^###^*p* < 0.001 vs. PBS/OVA group. Control, unexposed normal mice; PBS/OVA, asthmatic mice; 3d1μgLPS/OVA, low-dose (1 μg daily) LPS-exposed asthmatic mice with LPS exposure at 3rd DOL; 3d1μgLPS/PBS, low-dose (1 μg daily) LPS-exposed normal mice with LPS exposure at 3rd DOL.

Having demonstrated the inhibitory effects of low-dose LPS pre-exposure on TRIF/IRF3/IFNβ signaling in DCs and given the fact that IFNβ stimulates GITRL expression (21), we surmised that exogenous IFNβ exposure should rescue GITRL downregulation in low-dose LPS pre-exposed DCs. *In vitro*, primary CD11c^+^CD11b^+^ DCs and CD4^+^ T-cells were co-cultured with or without low-dose LPS pre-exposure in the presence of absence of IFNβ prior to OVA stimulation. By both flow cytometry and membrane-fraction immunoblotting, we found that IFNβ exposure completely rescued GITRL downregulation in low-dose LPS pre-exposed DCs (*p* < 0.05, Figures 6A,B).

In order to validate these findings *in vivo*, we employed the aforedescribed 3d1μgLPS/OVA murine model (both with and without IFNβ exposure during the LPS pre-exposure period) and assessed the changes in DC GITRL expression between control, PBS/OVA, 3d1μgLPS/OVA, and 3d1μgLPS/PBS mice. Consistent with our *in vitro* results, we found that IFNβ exposure completely rescued GITRL downregulation in 3d1μgLPS/OVA DCs (*p* < 0.05, Figures 6C,D). These combined findings reveal that the GITRL downregulation on DCs from low-dose LPS pre-exposure is attributable to decreased TRIF/IRF3/IFNβ signaling.

## Discussion

In this study, we hypothesized that LPS-induced changes in DC GITRL expression may impact Treg-mediated Th cell suppression and the induction of endotoxin tolerance. We found that low-dose LPS pre-exposure (1 μg) in neonatal asthmatic mice produces Treg skewing *via* promoting apoptosis of pathogenic Th2 and Th17 cells through downregulating DC GITRL expression. We also demonstrated that this DC GITRL downregulation is attributable to TLR4-dependent TRIF/IRF3/IFNβ signaling inhibition. These combined findings reveal that low-dose LPS pre-exposure produces tolerogenic Treg skewing in neonatal asthmatic mice, a phenomenon attributable to TLR4-dependent TRIF/IRF3/IFNβ-mediated DC GITRL downregulation.

In humans, the majority of asthma cases debut before the age of three with loss of lung function evident by 6 years of age (25). During these early years, environmental LPS exposure appears to play a critical role in either endotoxin tolerance or asthma development (4–6). Moreover, differing levels of LPS exposure can produce divergent immune responses in murine models of asthma (7). In order to construct an optimal murine model of endotoxin tolerance, here we applied various concentrations of LPS at different time points during the neonatal immune maturation period prior to OVA-induced asthma sensitization and challenge. We observed that low-dose (1 μg) LPS inhalation in 3-day old neonatal mice reduced AHR, peribronchial, and perivascular inflammation in lung tissues, serum OVA-specific IgE levels, and BALF levels of pro-eosinophilic/neutrophilic cytokines. On this basis, low-dose LPS pre-exposure in neonates appears to have a tolerogenic effect on later asthma development. Moreover, our findings suggest that variations in the exposure level and timing of environmental LPS exposure may explain the discordant phenotypes following LPS exposure.

Although the exact mechanism(s) underlying endotoxin tolerance in asthma remain unclear, current evidence suggests that T-cell distributions favoring Treg or Th1 cells over pathogenic Th2 and Th17 cells play important roles in the development of endotoxin tolerance (18). This is because Th2 cells produce the type 2 cytokines IL-4, IL-5, and IL-13 that drive eosinophilic inflammation and mucus production (26), while Th17 cell-produced IL-17 induces pathogenic smooth muscle contractions and structural alterations to the airway epithelium (27). Here, from both *in vivo* and *in vitro* experimentation, we found that low-dose LPS pre-exposure produces Treg skewing by promoting apoptosis of pathogenic Th2 and Th17 cells. Our findings are consistent with previous research demonstrating that Treg and/or Th1 skewing confers tolerogenic effects against asthma and other allergic disorders (28, 29).

The DC-expressed co-stimulatory molecule GITRL plays an important role in inhibiting Treg-mediated suppression of Th cells, thereby inhibiting tolerogenicity and eliciting autoimmune disease (16). Here, from both *in vivo* and *in vitro* experimentation, we found that low-dose LPS pre-exposure produces Treg skewing by promoting apoptosis of pathogenic Th2 and Th17 cells through downregulating DC GITRL expression. To confirm that GITRL downregulation was responsible for LPS’s tolerogenic effects, we silenced and overexpressed GITRL in lung-derived murine DCs and adoptively transferred these transfected DCs into mice. The adoptive transfer of GITRL-overexpressing DCs abrogated the tolerogenic effects of LPS pre-exposure as evidenced by increases in AHR, peribronchial, and perivascular inflammation in lung tissue, serum OVA-specific IgE levels, and BALF levels of pro-eosinophilic/neutrophilic cytokines. In contrast, the adoptive transfer of GITRL-silenced DCs produced the opposite effects. We validated these findings *in vitro via* co-culturing primary DCs with CD4^+^ T-cells. It is important to note that GITRL overexpression in adoptively transferred DCs did not completely inhibit Treg-mediated suppression of Th cells. This might reflect the activity of APCs other than DCs.

We next examined the molecular mechanism(s) underlying DC GITRL downregulation following low-dose LPS pre-exposure. Interestingly, although LPS acts through the Toll-like receptor TLR4 expressed on the surface of APCs, LPS pre-exposure has divergent downstream effects depending on the dosage of the initial LPS challenge (19, 20). While pre-exposure to low or high doses of LPS can induce a transient pro-inflammatory state followed by a refractory tolerant state (endotoxin tolerance), pre-exposure to super-low doses of LPS (picogram levels) produces a non-resolving inflammatory adaptation, a phenomenon Morris et al. terms endotoxin priming (19, 20). Current evidence suggests that the “switch” between endotoxin tolerance vs. endotoxin priming results from a complex competition between two TLR4-mediated signaling pathways in APCs, namely the MyD88-dependent IRAK/MAPK pathway and the MyD88-independent TRIF/IRF3 pathway (20). Specifically, while the MyD88-dependent IRAK/MAPK pathway induces pro-inflammatory NF-κB activation, it also activates multiple tolerogenic negative feedback loops (20). In contrast, the MyD88-independent TRIF/IRF3 pathway prevents endotoxin tolerance by downregulating the expression of tolerogenic negative regulators, such as SIKE and SARM (20, 22). Therefore, there is a competitive dynamic balance between the MyD88-dependent IRAK/MAPK pathway (favoring endotoxin tolerance) vs. the MyD88-independent TRIF/IRF3 pathway (favoring endotoxin priming) (20). Consistent with this molecular model, here we demonstrated that low-dose LPS pre-exposure inhibited TRIF/IRF3/IFNβ signaling and upregulated expression of the tolerogenic negative regulators SARM, PTPB1, and SIKE. Mechanistically, as SARM and PTPB1 interfere with TLR4-TRIF binding and SIKE suppresses TBK1 activation, SARM, PTPB1, and SIKE upregulation would synergistically act to inhibit TRIF/IRF3/IFNβ signal transduction (22, 30). More importantly, the observed tolerogenic DC GITRL downregulation is also attributable to this inhibition in TRIF/IRF3/IFNβ signaling, as IFNβ exposure completely abolished GITRL downregulation both *in vitro* and *in vivo*.

In conclusion, low-dose LPS pre-exposure (1 μg) produces tolerogenic Treg skewing in neonatal asthmatic mice, a phenomenon attributable to TLR4-dependent TRIF/IRF3/IFNβ-mediated DC GITRL downregulation. Our findings provide important cellular and molecular insights into the criticality of LPS exposure levels and timing in the development of endotoxin tolerance, which help explain the discordant conclusions regarding the effects of early environmental endotoxin exposure on later allergic responses. Moreover, our findings may provide guidance on the development of novel preventative approaches against asthma and other allergic disorders in young children *via* targeting the GITRL/GITR axis.

## Data Availability Statement

All datasets presented in this study are included in the article/Supplementary Material.

## Ethics Statement

The animal study was reviewed and approved by the Institutional Animal Care and Research Advisory Committee at Chongqing Medical University.

## Author Contributions

ZF, FD, BL conceived and designed the study. FD, BL, CN, TW, and YW performed the experiments. YW, DT, GG, and JD analyzed the data. FD and ZF drafted the manuscript. All authors contributed to the article and approved the submitted version.

## Conflict of Interest

The authors declare that the research was conducted in the absence of any commercial or financial relationships that could be construed as a potential conflict of interest.

## Abbreviations

AHR: airway hyperresponsiveness
APCs: antigen-presenting cells
DCs: dendritic cells
GITRL: glucocorticoid-induced tumor necrosis factor receptor ligand
IFNβ: interferon-β
IRF3: interferon regulatory factor 3
LPS: lipopolysaccharide
OVA: ovalbumin
TLR4: Toll-like receptor 4
Treg: regulatory T-cell
TRIF: TIR-domain-containing adapter-inducing interferon-β.
