# Supplementary material for: Low-Dose LPS Induces Tolerogenic Treg Skewing in Asthma
Source: Front Immunol. 2020 Sep 23;11:2150. doi: 10.3389/fimmu.2020.02150 (PMC7538595; doi:10.3389/fimmu.2020.02150)

**SUPPLEMENTARY FIGURE LEGENDS**

**Supplementary Figure 1. Experimental Protocol for Constructing Murine Model of Asthma**

Neonatal BALB/c mice received daily low-dose LPS (1 µg), high-dose LPS (100 µg), or PBS (control) intranasally (i.n.) from the 3^rd^ day of life (DOL) or 14^th^ DOL for a period of 10 consecutive days. Mice were then sensitized with 100 µg OVA plus 100 µl aluminum hydroxide (AlOH) or PBS (control) via intraperitoneal (i.p.) injection on the 42^nd^ DOL and 56^th^ DOL, and then further exposed to a daily, 30-min 1% OVA aerosol or PBS aerosol (control) from the 63^rd^ DOL for a period of 10 consecutive days. Twenty-four hours after the last OVA challenge, the mice were sacrificed. n = 6–8 mice per group.

**Supplementary Figure 2. Effects of the Various LPS Exposure Protocols on Mice Subjects**

(A) Lung resistance (LR) values in reaction to increasing doses of methacholine were measured 24 hours after the final challenge. (B) Representative images of lung tissue sections stained with hematoxylin and eosin (H&E) 24 hours after the final challenge. Magnifications: × 200 and × 400. (C) Serum OVA-specific IgE levels as measured by ELISA. n = 6–8 mice per group. Data are reported as means ± standard deviations (SDs). **p*<0.05, ***p*<0.01, and ****p*<0.001 vs. Control group; #*p*<0.05, ##*p*<0.01, and ###*p*<0.001 vs. PBS/OVA group. Abbreviations: Control, unexposed normal mice; PBS/OVA, asthmatic mice; 3d1µgLPS/OVA, low-dose (1 µg daily) LPS-exposed asthmatic mice with LPS exposure at 3^rd^ day of life (DOL); 3d1µgLPS/PBS, low-dose (1 µg daily) LPS-exposed normal mice with LPS exposure at 3^rd^ DOL; 3d100µgLPS/OVA, high-dose (100 µg daily) LPS-exposed asthmatic mice with LPS exposure at 3^rd^ DOL; 3d100µgLPS/PBS, high-dose (100 µg daily) LPS-exposed normal mice with LPS exposure at 3^rd^ DOL; 2w1µgLPS/OVA, low-dose (1 µg daily) LPS-exposed asthmatic mice with exposure at 14^th^ DOL; 2w1µgLPS/PBS, low-dose (1 µg daily) LPS-exposed normal mice with exposure at 14^th^ DOL; 2w100µgLPS/OVA, high-dose (100 µg daily) LPS-exposed asthmatic mice with LPS exposure at 14^th^ DOL; 2w100µgLPS/PBS, high-dose (100 µg daily) LPS-exposed normal mice with LPS exposure at 14^th^ DOL.

**Supplementary Figure 3. Comparison of T-Cell Profiles between Low-Dose LPS-Exposed Asthmatic Mice and Untreated Asthmatic Mice**

(A) Profiling of Treg (CD4^+^CD25^+^Foxp3^+^) cells, Th1 (CD4^+^INF-γ^+^) cells, Th2 (CD4^+^IL-4^+^) cells, and Th17 (CD4^+^IL17^+^) cells in the lungs by flow cytometry. The proportions of Th2 and Th17 T-cells were significantly decreased in low-dose LPS-exposed asthmatic mice (Th2: 0.36±0.09 vs 1.04±0.12 vs 0.61±0.08 vs 0.33±0.07, Th17: 0.96±0.12 vs 2.51±0.15 vs 1.62±0.11 vs 1.02±0.07), while the number and function of Treg cells were significantly increased low-dose LPS-exposed asthmatic mice (Treg: 10.38±2.03 vs 6.76±1.36 vs 14.21±1.84 vs 10.21±1.92). (B) Quantitative real-time reverse transcription PCR (qRT-PCR) analysis of Treg/Th1/Th2/Th17-associated transcription factor mRNA expression (Foxp3, T-bet, GATA3, and ROR-γt, respectively) in T-cell subsets sorted from lung by flow-cytometry. (C) Treg/Th1/Th2/Th17-associated cytokine levels in bronchoalveolar lavage fluid (BALF) as measured by ELISA. n = 6–8 mice per group. Each experiment was repeated five times. Data are reported as means ± standard deviations (SDs). **p*<0.05, ***p*<0.01, and ****p*<0.001 vs. Control group; #*p*<0.05, ##*p*<0.01, and ###*p*<0.001 vs. PBS/OVA group. Abbreviations: Control, unexposed normal mice; PBS/OVA, asthmatic mice; 3d1µgLPS/OVA, low-dose (1 µg daily) LPS-exposed asthmatic mice with LPS exposure at 3^rd^ day of life (DOL); 3d1µgLPS/PBS, low-dose (1 µg daily) LPS-exposed normal mice with LPS exposure at 3^rd^ DOL.

**Supplementary Figure 4. Apoptosis Levels of Low-Dose LPS-Exposed T-Cells Co-Cultured with Dendritic Cells Prior to OVA Stimulation**

Low-dose LPS exposure significantly increased apoptosis levels of Th2 and Th17 cells but produced no significant changes in Treg or Th1 cell apoptosis levels. Cleaved caspase-3 expression and apoptosis levels of (A) Treg, (B) Th1, (C) Th2, and (D) Th17 cells. Data are reported as means ± standard deviations (SDs). **p*<0.05, ***p*<0.01, and ****p*<0.001 vs. Control group; #*p*<0.05, ##*p*<0.01, and ###*p*<0.001 vs. PBS/OVA group. Abbreviations: Control, unexposed normal mice; PBS/OVA, asthmatic mice; LPS/OVA, low-dose LPS-exposed asthmatic mice; LPS/PBS, low-dose LPS-exposed normal mice.

**Supplementary Figure 5. Lung-Derived T-Cell GITR Expression Downregulated in Low-Dose LPS-Exposed Asthmatic Mice Relative to Untreated Asthmatic Mice**

Surface GITR expression in (A) Treg (CD4^+^CD25^+^Foxp3^+^) cells, (B) Th1 (CD4^+^INF-γ^+^) cells, (C) Th2 (CD4^+^IL-4^+^) cells, and (D) Th17 (CD4^+^IL-17^+^) cells was significantly downregulated in low-dose LPS-exposed asthmatic mice relative to untreated asthmatic mice. Abbreviations: Control, unexposed normal mice; PBS/OVA, asthmatic mice; 3d1µgLPS/OVA, low-dose (1 µg daily) LPS-exposed asthmatic mice with LPS exposure at 3^rd^ day of life (DOL); 3d1µgLPS/PBS, low-dose (1 µg daily) LPS-exposed normal mice with LPS exposure at 3^rd^ DOL.

**Supplementary Figure 6. Co-Culture of Low-Dose LPS-Exposed Dendritic Cells and T-Cells Prior to OVA Stimulation Downregulates T-Cell GITR Expression**

Surface GITR expression on (A) Treg (CD4^+^CD25^+^Foxp3^+^) cells, (B) Th1 (CD4^+^INF-γ^+^) cells, (C) Th2 (CD4^+^IL-4^+^) cells, and (D) Th17 (CD4^+^IL17^+^) cells was significantly downregulated in the low-dose LPS-exposed cells as compared with LPS/OVA cells. Abbreviations: Control, unexposed normal mice; PBS/OVA, asthmatic mice; LPS/OVA, low-dose LPS-exposed asthmatic mice; LPS/PBS, low-dose LPS-exposed normal mice.

**Supplementary Figure 7. Validation of Altered GITRL Transcript Expression in Lung-Derived Dendritic Cells Following Delivery of GITRL siRNA or GITRL Overexpression Plasmid**

qRT-PCR of GITRL transcript expression in lung-derived dendritic cells. Data are reported as means ± standard deviations (SDs). **p*<0.05, ***p*<0.01, and ****p*<0.001 vs. Control group; #*p*<0.05, ##*p*<0.01, and ###*p*<0.001 vs. 3d1µgLPS/OVA group. Abbreviations: Control, unexposed normal mice; Control+Vector, unexposed normal mice with empty vector; PBS/OVA, asthmatic mice; 3d1µgLPS/OVA, low-dose LPS-exposed asthmatic mice; 3d1µgLPS/OVA+GITRL-siRNA, low dose LPS-exposed asthmatic mice with GITRL-siRNA dendritic cells; 3d1µgLPS/OVA+GITRL-OE, low dose LPS-exposed asthmatic mice with GITRL-overexpressing dendritic cells.

**Supplementary Figure 8. Effects of DC GITRL Silencing or Overexpression on T-Cells from Low-Dose LPS-Exposed Asthmatic Mice**

(A) Profiling of Treg (CD4^+^CD25^+^Foxp3^+^), Th1 (CD4^+^INF-r^+^), Th2 (CD4^+^IL-4^+^), and Th17 (CD4^+^IL17^+^) T-cells by flow cytometry. (B) Quantitative real-time reverse transcription PCR (qRT-PCR) analysis of Treg/Th1/Th2/Th17-associated transcription factor mRNA expression (Foxp3, T-bet, GATA3, ROR-γt, respectively) in flow-cytometry sorted T-cell subsets. (C) Treg/Th1/Th2/Th17-associated cytokine levels in bronchoalveolar lavage fluid (BALF) as measured by ELISA. n = 6–8 mice per group. Data are reported as means ± standard deviations (SDs). **p*<0.05, ***p*<0.01, and ****p*<0.001 vs. PBS/OVA group; #*p*<0.05, ##*p*<0.01, and ###*p*<0.001 vs. 3d1µgLPS/OVA group. Abbreviations: Control, unexposed normal mice; Control+Vector, unexposed normal mice with empty vector; PBS/OVA, asthmatic mice; 3d1µgLPS/OVA, low-dose LPS-exposed asthmatic mice; 3d1µgLPS/OVA+GITRL-siRNA, low dose LPS-exposed asthmatic mice with GITRL-siRNA dendritic cells; 3d1µgLPS/OVA+GITRL-OE, low dose LPS-exposed asthmatic mice with GITRL-overexpressing dendritic cells.

**Supplementary Figure 9. Validation of Altered GITRL Transcript Expression in Cultured Dendritic Cells Following Delivery of GITRL siRNA or GITRL Overexpression Plasmid**

qRT-PCR of GITRL transcript expression in cultured dendritic cells. Data are reported as means ± standard deviations (SDs). **p*<0.05, ***p*<0.01, and ****p*<0.001 vs. Control group; #*p*<0.05, ##*p*<0.01, and ###*p*<0.001 vs. LPS/OVA group. Abbreviations: Control, CD11c^+^CD11b^+^ dendritic cells plus CD4^+^ T-cells; Control+Vector, CD11c^+^CD11b^+^ -empty vector dendritic cells plus CD4^+^ T-cells; PBS/OVA, CD11c^+^CD11b^+^ dendritic cells plus CD4^+^ T-cells treated with OVA; LPS/OVA, low dose LPS-exposed CD11c^+^CD11b^+^ dendritic cells plus CD4^+^ T-cells treated with OVA; LPS/OVA+GITRL-siRNA, low dose LPS-exposed CD11c^+^CD11b^+^-GITRL-silenced dendritic cells plus CD4^+^ T-cells treated with OVA; LPS/OVA+GITRL-OE, low dose LPS-exposed CD11c^+^CD11b^+^-GITRL-overexpressing dendritic cells plus CD4^+^ T-cells treated with OVA.

**Supplementary Figure 10. Effects of GITRL Silencing or Overexpression on Low-Dose LPS-Exposed Dendritic Cells Co-Cultured with T-Cells Prior to OVA Stimulation**

(A) Profiling of Treg (CD4^+^CD25^+^Foxp3^+^), Th1 (CD4^+^INF-r^+^), Th2 (CD4^+^IL-4^+^), and Th17 (CD4^+^IL17^+^) T-cells by flow cytometry. (B) Quantitative real-time reverse transcription PCR (qRT-PCR) analysis of Treg/Th1/Th2/Th17-associated transcription factor mRNA expression (Foxp3, T-bet, GATA3, ROR-γt, respectively) in flow-cytometry sorted T-cell subsets. (C) Treg/Th1/Th2/Th17-associated cytokine levels as measured by ELISA. n = 6–8 mice per group. Data are reported as means ± standard deviations (SDs). **p*<0.05, ***p*<0.01, and ****p*<0.001 vs. PBS/OVA group; #*p*<0.05, ##*p*<0.01, and ###*p*<0.001 vs. LPS/OVA group. Abbreviations: Control, CD11c^+^CD11b^+^ dendritic cells plus CD4^+^ T-cells; Control+Vector, CD11c^+^CD11b^+^ -empty vector dendritic cells plus CD4^+^ T-cells; PBS/OVA, CD11c^+^CD11b^+^ dendritic cells plus CD4^+^ T-cells treated with OVA; PBS/OVA+GITRL-siRNA, CD11c^+^CD11b^+^-GITRL-siRNA dendritic cells plus CD4^+^ T-cells treated with OVA; LPS/OVA, low dose LPS-exposed CD11c^+^CD11b^+^ dendritic cells plus CD4^+^ T-cells treated with OVA; LPS/OVA+GITRL-siRNA, low dose LPS-exposed CD11c^+^CD11b^+^-GITRL-siRNA dendritic cells plus CD4^+^ T-cells treated with OVA; LPS/OVA+GITRL-OE, low dose LPS-exposed CD11c^+^CD11b^+^-GITRL dendritic cells plus CD4^+^ T-cells treated with OVA.

**Supplementary Figure 11. Low-Dose LPS Pre-Exposure Dysregulates TLR4-Mediated IRF3 Dimerization, Nuclear Translocation, and Transactivation in Dendritic Cells**

TLR4-mediated IRF3 dimerization, nuclear translocation, and transactivation in CD11c^+^CD11b^+^ dendritic cells were significantly downregulated in the low-dose LPS-exposed LPS/OVA cells (100 ng/ml LPS) as compared with PBS/OVA cells. (A) Cytoplasmic-fraction immunoblotting of IRF3 dimer and IRF3 monomer expression in primary dendritic cells. (B) Nuclear-fraction immunoblotting of p-IRF3 and IRF3 expression in primary dendritic cells. (C) A dual luciferase reporter assay was performed in primary dendritic cells to assess IRF3 transactivation potential.

**SUPPLEMENTARY FIGURES**

**Supplementary Figure 1**


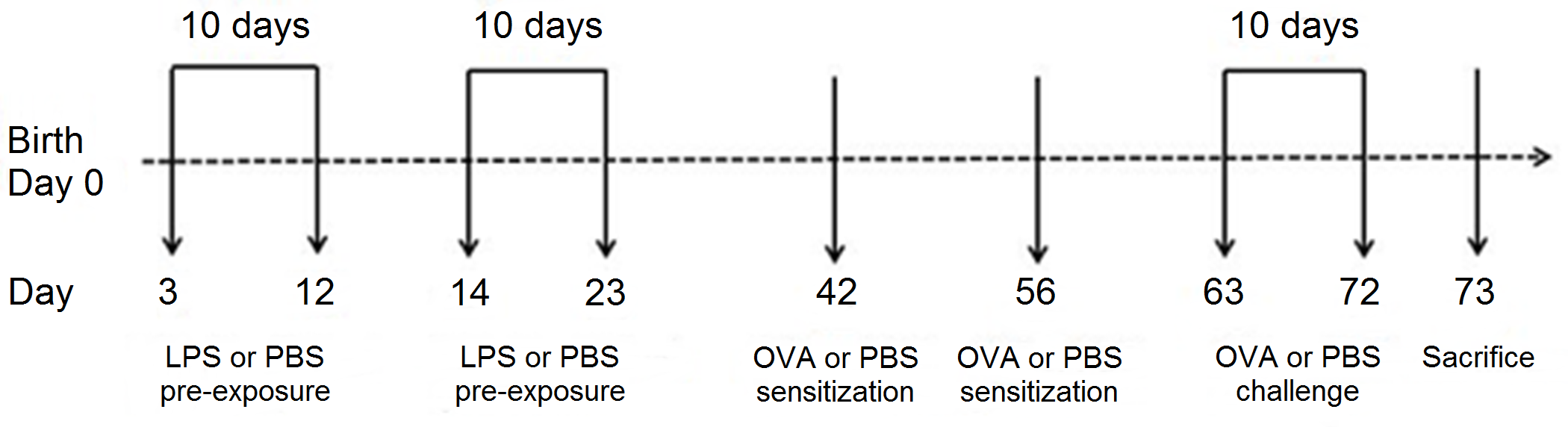


**Supplementary Figure 2**


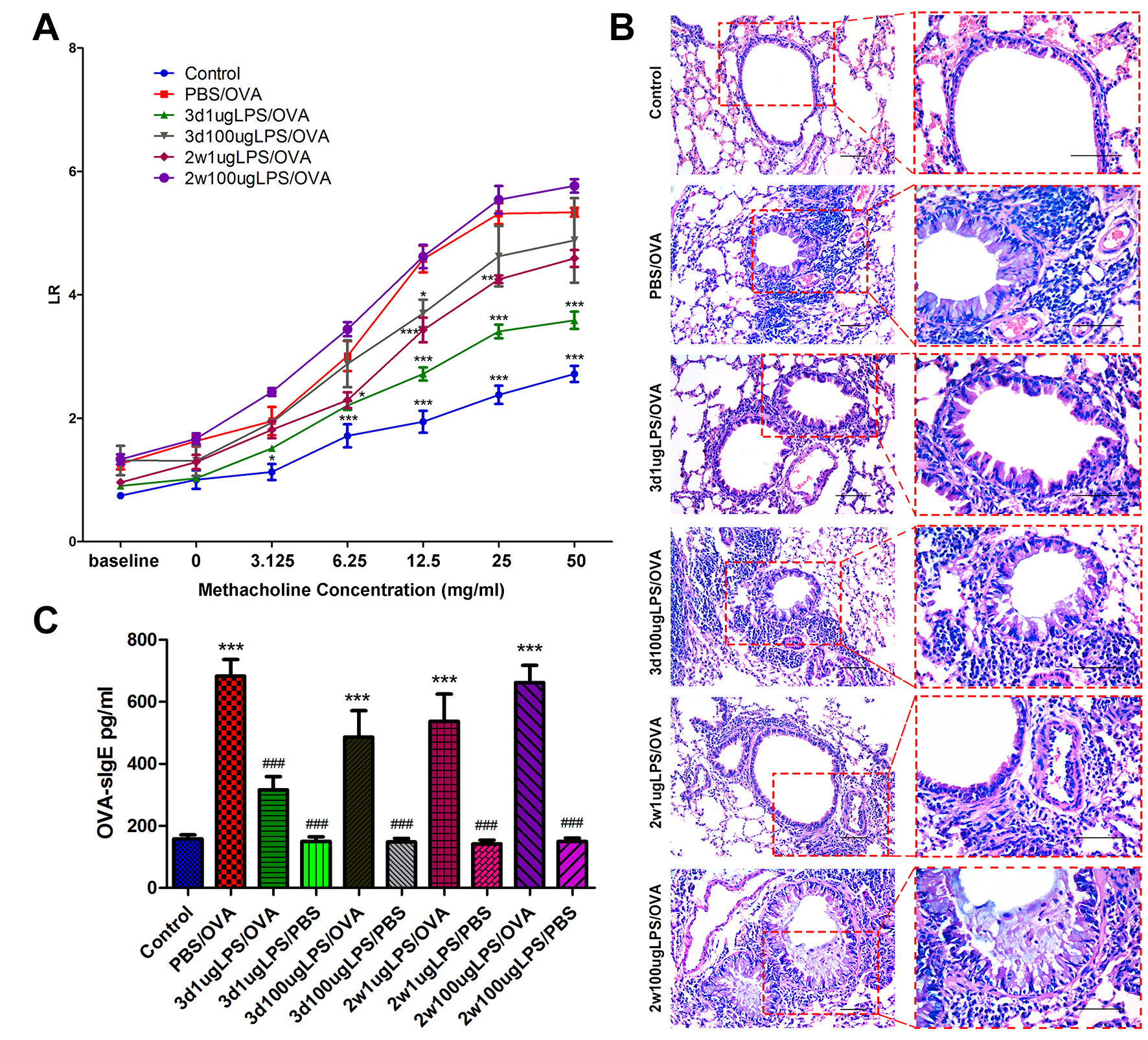


**Supplementary Figure 3**


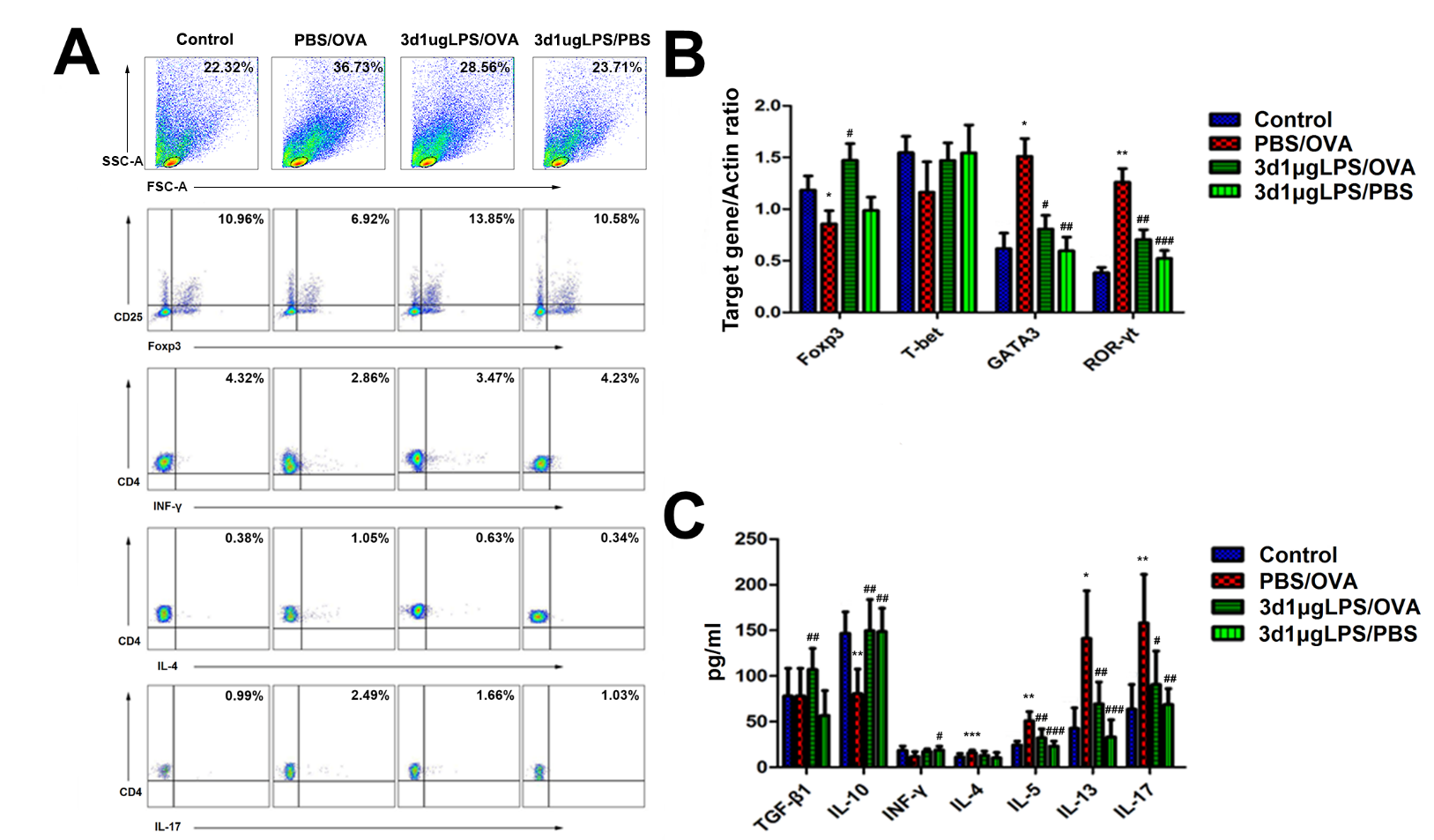


**Supplementary Figure 4**


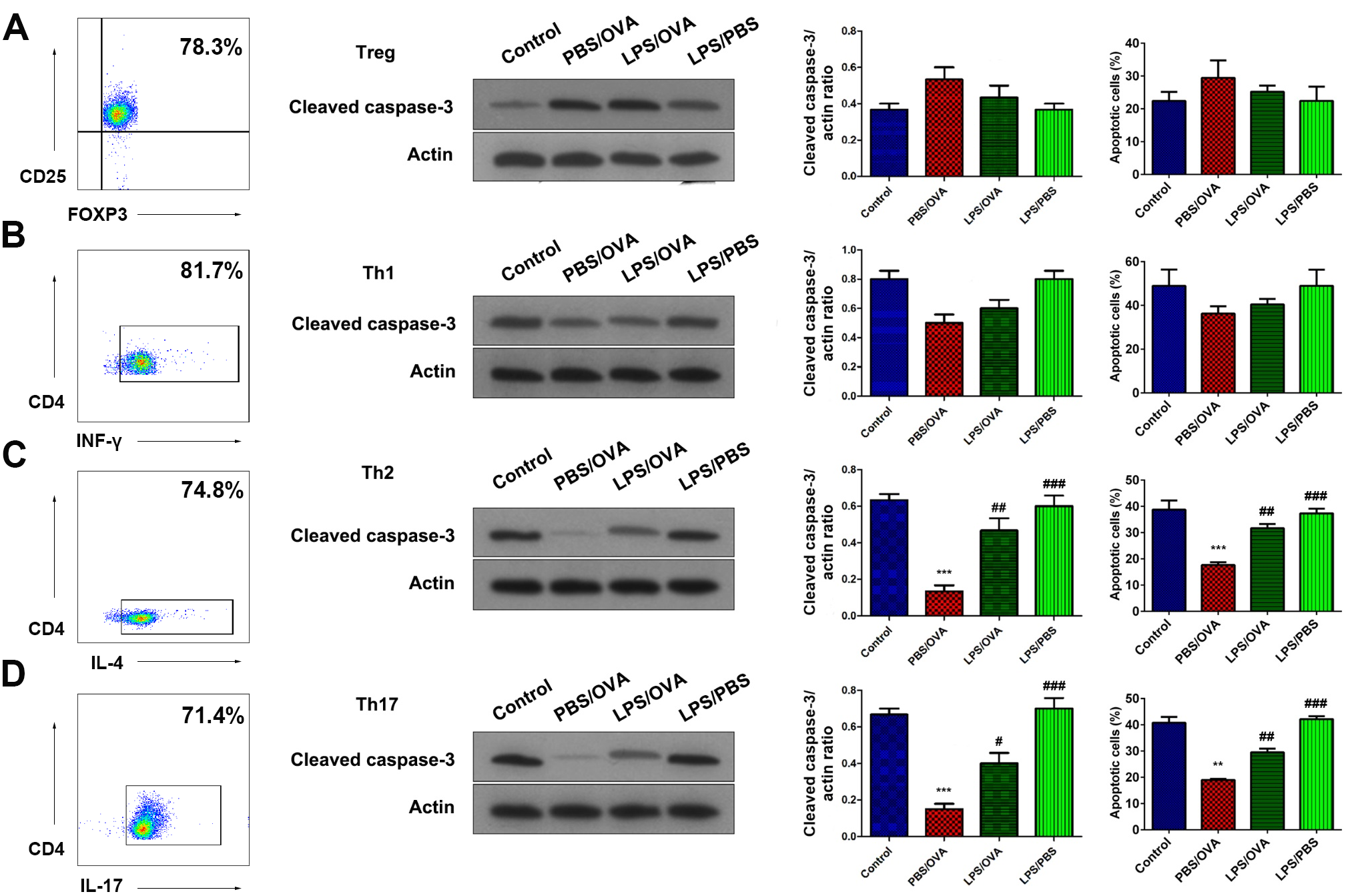


**Supplementary Figure 5**


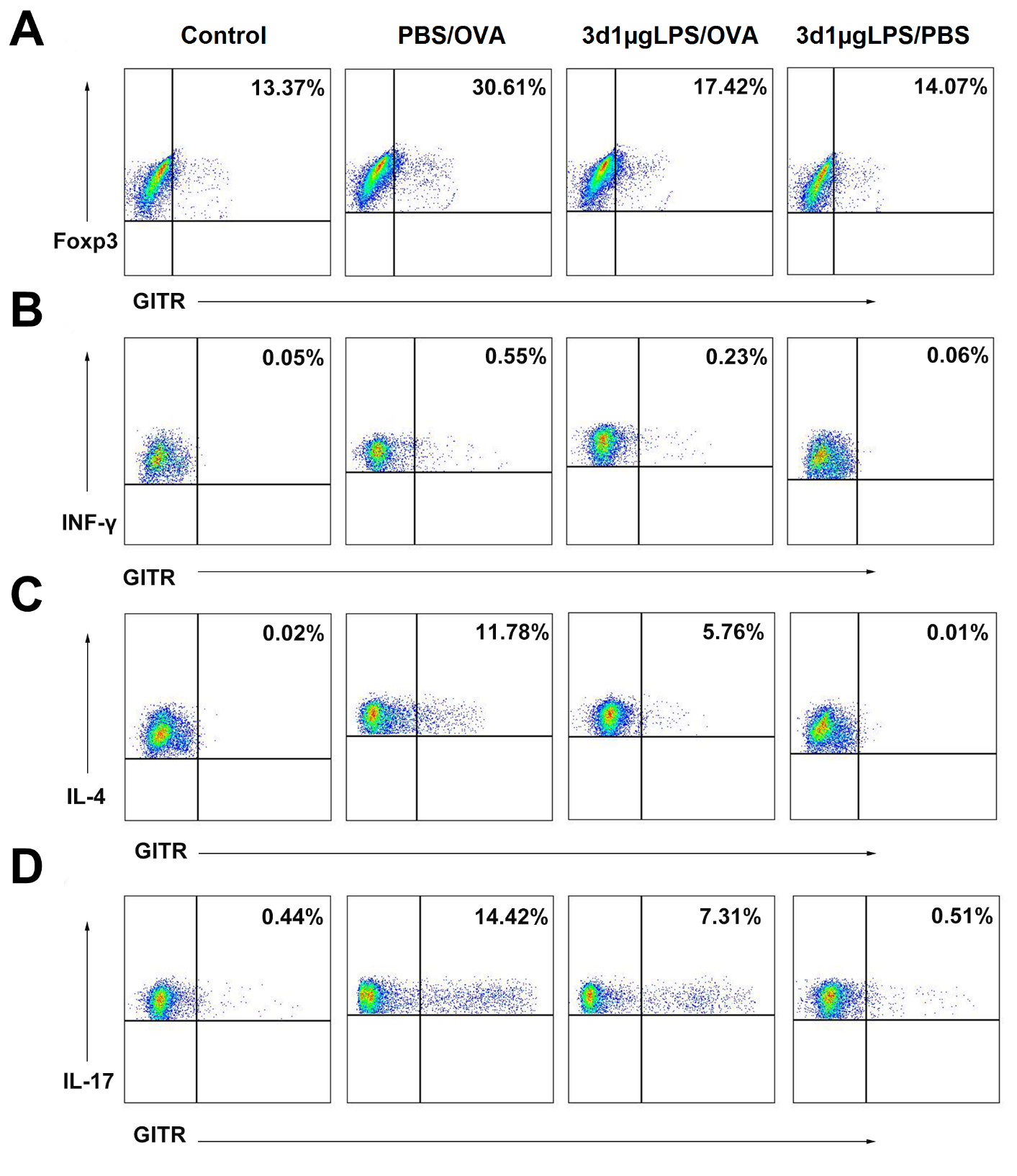


**Supplementary Figure 6**


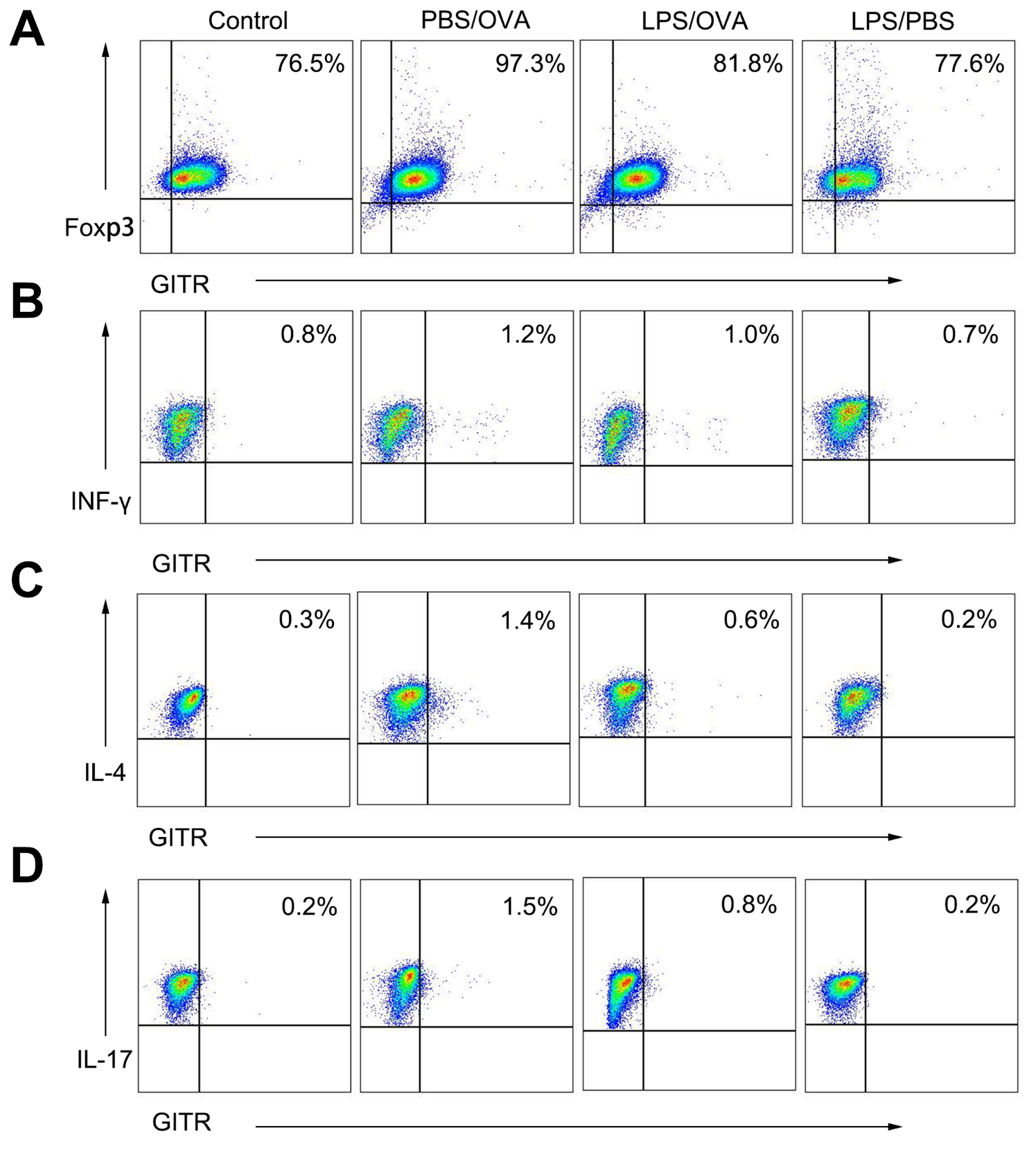


**Supplementary Figure 7**


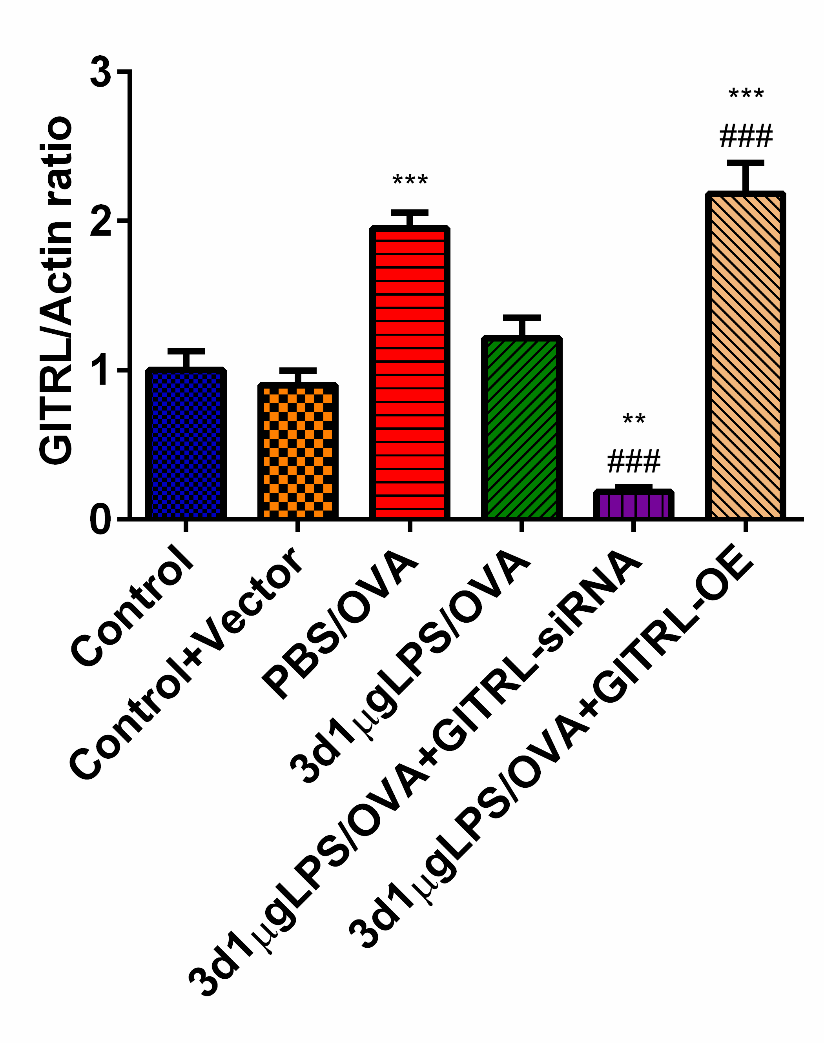


**Supplementary Figure 8**


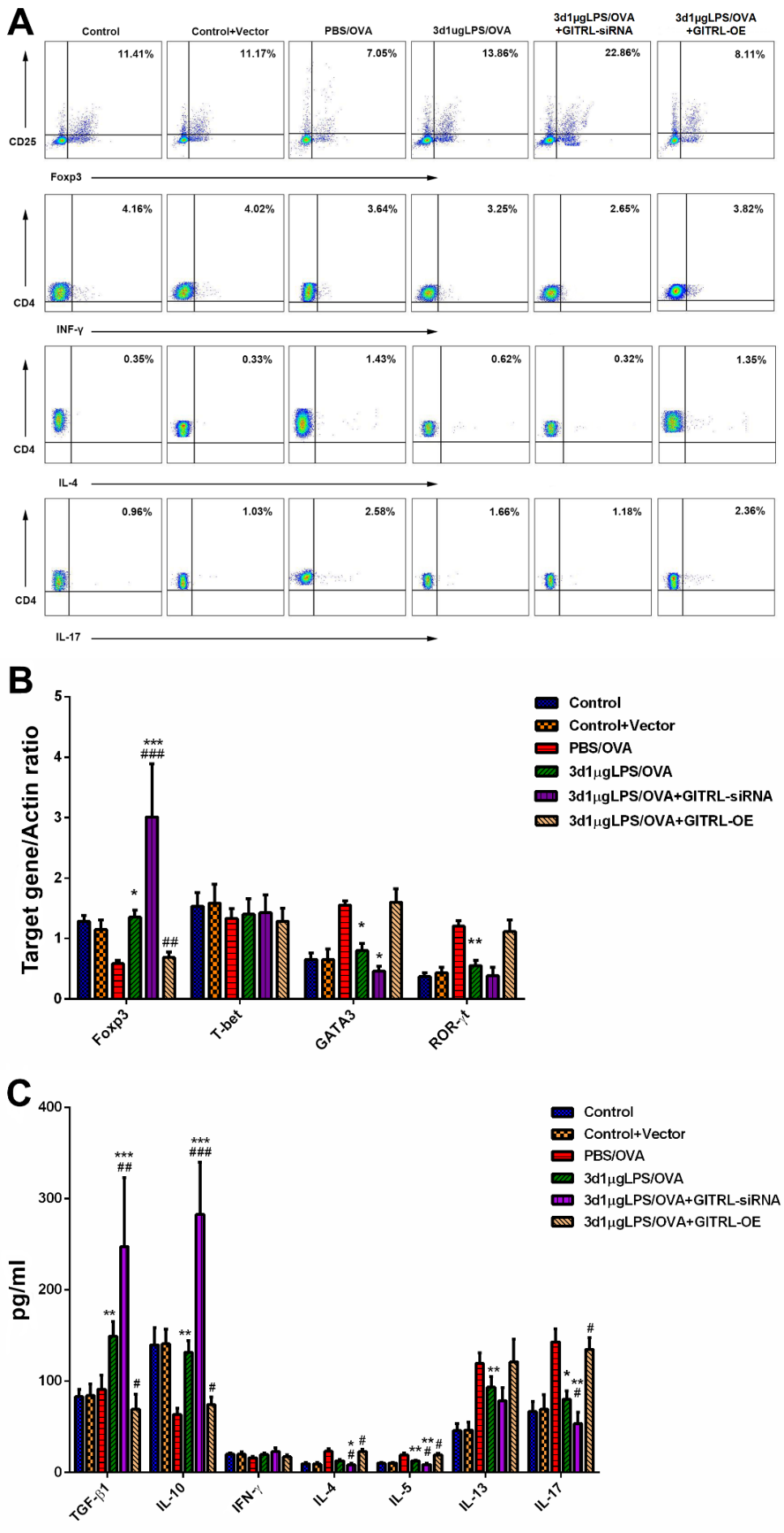


**Supplementary Figure 9**


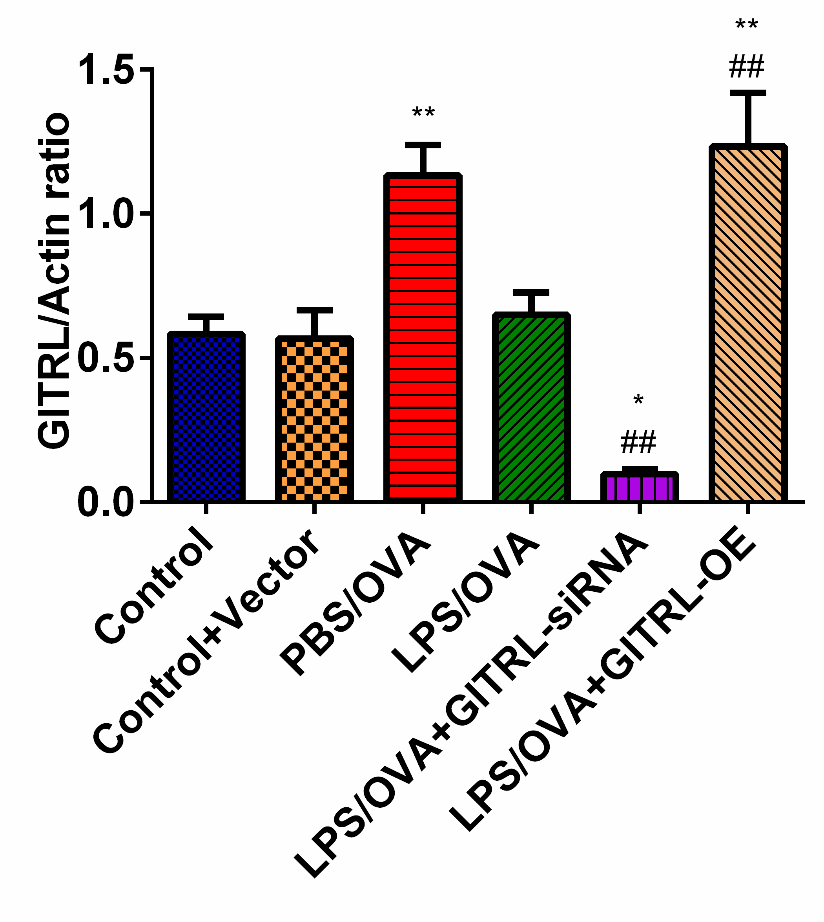


**Supplementary Figure 10**


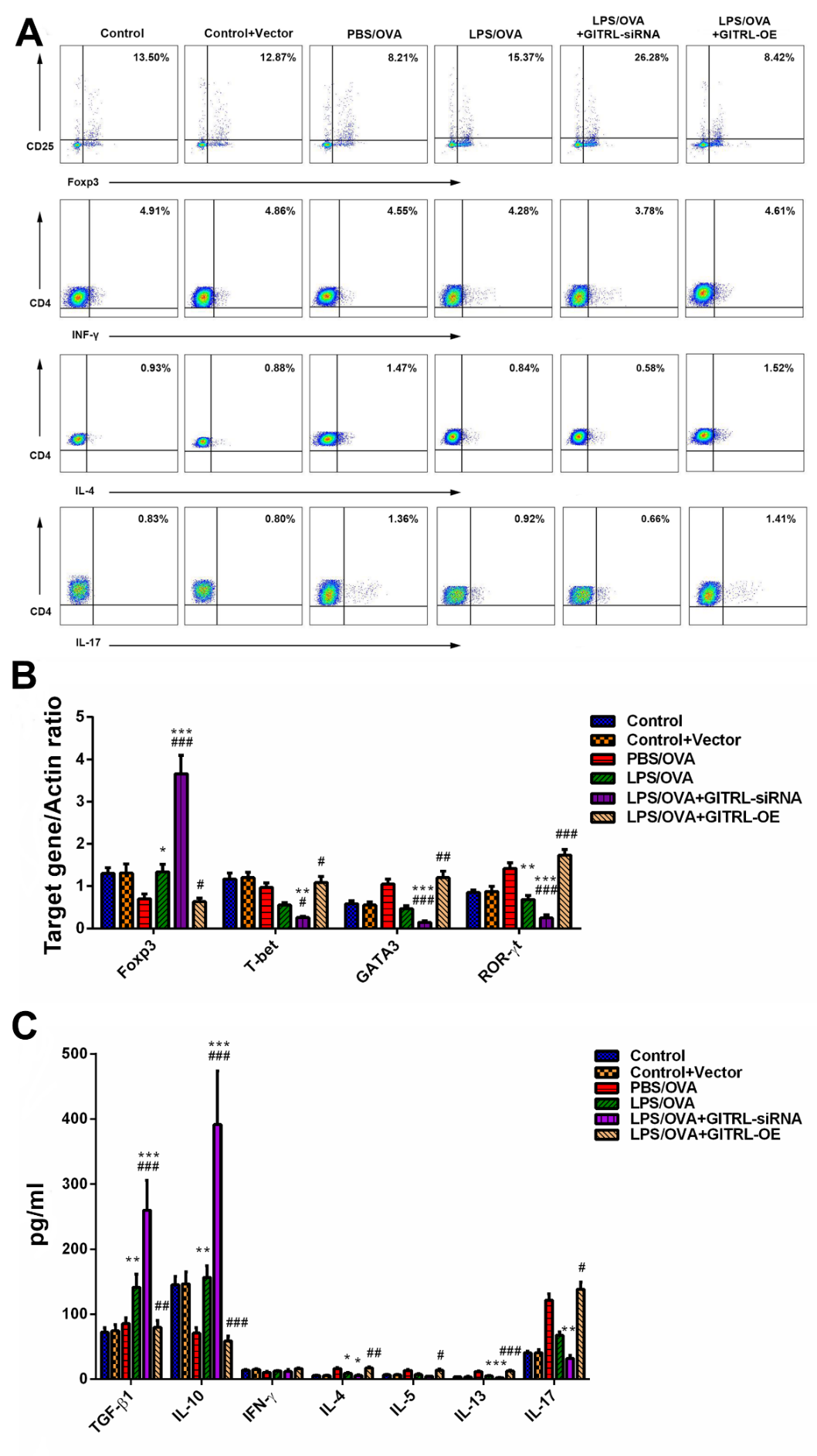


**Supplementary Figure 11**


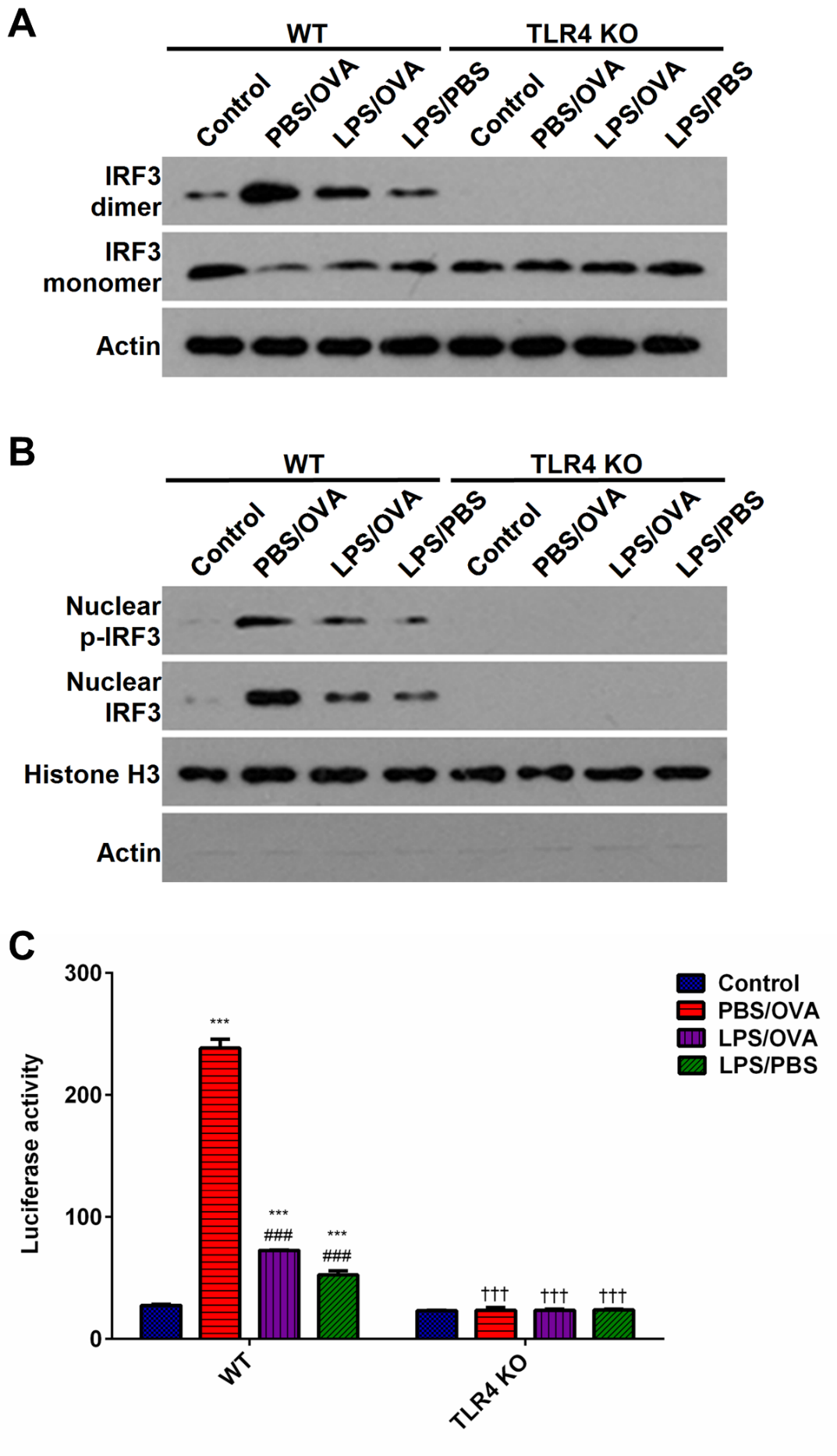

Supplement: Supplementary file 1 [file Data_Sheet_1.docx]
