# Supplementary material for: Low-Dose LPS Induces Tolerogenic Treg Skewing in Asthma
Source: Front Immunol. 2020 Sep 23;11:2150. doi: 10.3389/fimmu.2020.02150 (PMC7538595; doi:10.3389/fimmu.2020.02150)
Supplement: Supplementary file 2 [file Data_Sheet_2.docx]

**SUPPLEMENTARY INFORMATION FOR**

**Low-Dose LPS Induces Tolerogenic Treg Skewing in Asthma**

**MATERIALS AND METHODS**

**Animals**

Pathogen-free neonatal wild-type (WT) and Toll-like receptor 4 knockout (TLR4 KO) BALB/c mice were obtained from the Animal Breeding Facility at Chongqing Medical University (Chongqing, China). The neonates were housed with their mothers in individually-filtered cages, maintained on a 12 h light/dark cycle with an ovalbumin (OVA)-free diet under a constant room temperature (24°C). Adequate amounts of sterile animal food and water were provided. Cages, food, bedding, and water were sterilized before use. The Institutional Animal Care and Research Advisory Committee at Chongqing Medical University (Chongqing, China) approved the protocols of this study. The use of animals in these experiments was in accordance with the guidelines issued by the Chinese Council on Animal Care. Commercial LPS was handled in a biohazard safety equipment.

**Construction of OVA Murine Model of Asthma**

A schematic describing the construction of the OVA murine model of asthma is provided in Supplementary Figure 1. Ultrapure LPS (Escherichia coli serotype 0111:B4; Sigma, Saint Louis, MO, USA) was repurified according to Hirschfeld et al.’s protocol ^1^. Neonatal mice were exposed to LPS by daily intranasal application of 1 µg or 100 µg LPS dissolved in 10 µl sterile phosphate buffered saline (PBS) for a consecutive period of 10 days starting on the 3^rd^ or 14^th^ day of life (DOL). Control animals received an equivalent volume of sterile PBS in an identical manner. In some experiments, neonatal mice were exposed to subcutaneous murine recombinant interferon-β (IFNβ) (5000 U, Hycult Biotech, Uden, The Netherlands) daily concurrently with LPS or PBS ^2^.

After LPS exposure, the OVA-induced murine asthma model was established on the sixth week of life according to the standard protocols ^3^. Briefly, mice were sensitized with 100 µg OVA (Sigma) plus 100 µl aluminum hydroxide (AlOH) or PBS (control) via intraperitoneal (i.p.) injection on the 42^nd^ DOL and 56^th^ DOL, and then further exposed to a daily, 30-min 1% OVA aerosol or PBS aerosol (control) challenge from the 63^rd^ DOL for a period of 10 consecutive days.

**Assessment of AHR**

Twenty-four hours after the final OVA challenge, lung resistance (LR) was measured using an invasive lung function test ^4^. Briefly, anesthetized mice were intubated and mechanically ventilated by a computer-controlled piston ventilator (Flexivent, SciReq, Montreal, Canada). Mice were then challenged with the bronchoconstrictor acetyl-β-methylcholine (Sigma) at increasing doses as follows: 0, 3.125, 6.25, 12.5, 25, and 50 mg/ml. At each dose, LR was calculated using the linear single compartment model ^5^.

**Bronchoalveolar Lavage and Leukocyte Counts**

Twenty-four hours after the final OVA challenge, mice were sacrificed, the trachea was cannulated, and bronchoalveolar lavage fluid (BALF) was obtained by flushing the lungs three times with 1.5 ml PBS. Total leukocyte numbers were counted in the BALF using a hemocytometer. Differential cell counts were performed under Wright-Giemsa staining and based on standard morphologic and staining characteristics of at least 200 cells per sample. All slides were evaluated by a single-blinded examiner to reduce evaluator bias. The remaining BALF was stored at -80°C for later analysis.

**Histological Analysis**

Twenty-four hours after the final OVA challenge, mice were sacrificed to harvest the lungs. Formalin-fixed lungs were embedded in paraffin. The paraffin blocks were serially-sectioned at a 4-micron thickness and stained with hematoxylin-eosin (H&E) or processed for immunohistochemical (IHC) analysis. Antibodies against GITRL (1:200; GeneTex, Irvine, CA, USA) were used for IHC staining. Lung tissue samples staining light yellow-to-brown were considered positive. The intensities of the GITRL IHC staining signal in BALB/c mice with different treatments were quantified using the Image-Pro Plus 6.0 image analysis software (Media Cybernetics, Inc. Silver Spring, MD, USA). Images were captured under a Nikon Eclipse E200 microscope connected to a Nikon Coolpix 995 camera (Nikon, Tokyo, Japan). Inflammation score was scored on a 0-3 scale defined as: 0-no inflammatory reaction; 1-mild inflammation with foci of inflammatory cells in bronchial or vascular wall and in alveolar septa; 2-moderate inflammation with patchy inflammation or localized inflammation in walls of bronchi or blood vessels and alveolar septa, and less than one-third of lung cross-sectional area is involved; and 3-severe inflammation with diffuse inflammatory cells in walls of bronchi or blood vessels and alveoli septa; between one-third and two-thirds of the lung area is involved.

**Isolation of Murine Lung-Derived DCs and T-Cells**

Lungs were minced and incubated for 20 min at 37°C in 1 ml of sterile PBS containing 0.2% collagenase I (Sigma). Single pulmonary cell suspensions were obtained by forcing tissue through a 70-μm cell filter (Becton, Dickinson and Company, Franklin Lakes, NJ, USA). Erythrocytes were lysed, and the remaining cells were resuspended in RPMI 1640 medium containing 10% fetal bovine serum (FBS). The single-cell suspension (2×10^6^ cells/ml) was incubated for 4–6 h at 37°C and 5% CO_2_ in six-well flat-bottom plates (Nalgene, Waltham, MA, USA) in 1 ml medium containing phorbol 12-myristate 13-acetate (50 ng/ml; Sigma), ionomycin (500 ng/mL; Sigma) and GolgiPlug-containing brefeldin A (Becton, Dickinson and Company). The cells were then harvested, washed, pretreated with an Fc blocker, and subsequently stained as follows.

To detect and isolate conventional CD11c^+^CD11b^+^ DCs and avoid contamination with other cells expressing CD11c and CD64 (e.g., alveolar macrophages), the following multi-tiered isolation strategy was employed as previously described ^6^. Briefly, CD11c^+^ cells were positively selected by MACS isolation using CD11c^+^ magnetic beads. These enriched CD11c^+^ cells were then stained for surface-associated CD64 (anti-CD64-PE; BD Pharmingen), MHCII (anti-MHCII-FITC; BD Pharmingen), CD11b (anti-CD11b-Alexa Fluor 488; BD Pharmingen), and CD11c (anti-CD11c-phycoerythrin/Cy7; BD Pharmingen). After outgating dead cells, doublets, and debris using MHCII as the *x*-axis, CD64 was plotted against MHCII, and all CD64^+^ cells were gated out. MHCII^+^CD11c^+^ cells were gated in, divided into CD11b^+^ and CD11b^-^ cells, and CD11c^+^CD11b^+^ DCs were finally selected by gating out CD11b^-^ cells. The purity of the FACS-sorted CD11c^+^CD11b^+^ DCs as assessed by flow cytometry was >95%.

To detect the subsets of CD4^+^ T-cells, the cells were stained for surface-associated CD3 (anti-CD3-PerCP-Cy5.5; BD Pharmingen, San Diego, CA, USA), CD4 (anti-CD4-FITC; BD Pharmingen), CD25 (anti-CD25-PE; BD Pharmingen), intracellular IFN-γ (anti-IFN-γ-PE-Cy7; BD Pharmingen), IL-17A (anti-IL-17A-PE; BD Pharmingen), IL-4 (anti-IL-4-APC; BD Pharmingen), and FOXP3 (anti-FOXP3-APC; BD Pharmingen).

Flow cytometry was performed on a fluorescence-activated cell sorter (FACS) Canto (Becton, Dickinson and Company). The data were analyzed with Cell Quest software (Becton, Dickinson and Company).

**Isolation of Murine Bone Marrow-Derived DCs**

After removing all muscle tissues with gauze from the femurs and tibias, the bones were transferred into a fresh dish with RPMI 1640 medium. Both ends of the bones were cut with scissors in the dish, and then the marrow was ﬂushed out using 3 ml RPMI 1640 medium with a syringe. The tissue was suspended and passed through nylon mesh to remove small pieces of bone and debris. Then, the bone marrow-derived mononuclear cells were cultured at a density of 2×10^6^ cells/ml in RPMI 1640 medium supplemented with 10% fetal calf serum (Gibco, Waltham, MA, USA), 10 ng/ml recombinant GM-CSF, and 5 ng/ml recombinant IL-4 at 37˚C. Non-adherent cells were gently washed away after 48 h of culture. CD11c^+^CD11b^+^ DCs were then isolated by FACS as described earlier. The purity of the FACS-sorted CD11c^+^CD11b^+^ DCs as assessed by flow cytometry was >95%.

**Artificial GITRL Overexpression and Silencing in Bone Marrow-Derived DCs**

Artificial GITRL overexpression was conducted using a murine cytomegalovirus vector (mCMV) as previously described with minor modifications ^7^. Briefly, the entire GITRL open reading frame (ORF) ^8^ was PCR-amplified and cloned into the pDC316-mCMV-EGFP shuttle vector in order to generate pDC316-mCMV-GITRL-EGFP. An empty pDC316-mCMV-EGFP shuttle vector was used as a transfection control. Viral particles were amplified through cotransfecting HEK293 cells in serum-free RPMI 1640 medium with pDC316-mCMV-CCR7-EGFP or pDC316-mCMV-EGFP and the adenovirus genomic plasmid pBHGloxΔE1, 3Cre with Lipofectamine 2000 (Promega, Madison, WI, USA). The transfected HEK293 cells were then incubated for seven days at 37°C and finally lysed with three freeze/thaw cycles. By centrifugation, the crude recombinant viral particles were collected from the supernatant and then subjected to three plaque purification rounds in HEK293 cells. GITRL and control adenoviral titers were quantified by qRT-PCR. Finally, 1×10^6^ bone marrow-derived DCs were mixed with 5×10^7^ plaque-forming units (PFUs) of either pDC316-mCMV-CCR7-EGFP or pDC316-mCMV-EGFP in a total volume of 1 ml serum-free RPMI 1640 medium for 24 h.

The silencing of GITRL mRNA expression was performed as described earlier with minor modifications ^9^. Briefly, bone marrow-derived DCs in serum-free RPMI 1640 medium were transfected with a GITRL-small interfering (si)RNA (Santa Cruz Biotechnology) or a control scrambled siRNA (Santa Cruz Biotechnology) to a final concentration of 100 nM using Lipofectamine 2000. Fresh RPMI-1640 medium containing 10% FBS was added 6 h after transfection, and the cells were then cultured for 48 h at 37°C. For validation, surface and membrane GITRL expression were determined by flow cytometry (anti-GITRL-PE; BD Pharmingen) and membrane-fraction Western blotting, respectively.

**Adoptive Transfer of Bone Marrow-Derived DCs into Mice**

Adoptive transfer of bone marrow-derived CD11c^+^CD11b^+^ DCs was performed as previously described with minor modifications ^10^. Briefly, freshly-purified DCs were collected and intravenously injected (1 × 10^6^ DCs in 200 μl) five days before the first OVA sensitization. The control group was injected with PBS alone (instead of OVA, as described earlier), while the control+vector group received DCs with an empty pDC316-mCMV-EGFP shuttle vector.

**Isolation of Murine Splenic T-Cells**

Single-cell suspensions of T-cells were prepared from whole mouse spleens. Peripheral blood mononuclear cells were isolated by density centrifugation over Lymphoprep (Axis-Shield, Oslo, Norway). CD4^+^ T-cells were then isolated by negative selection with a CD4-negative Cell Isolation Kit (Miltenyi Biotec) using an AutoMACS cell sorter (Miltenyi Biotec) and a double-column magnetic separation procedure as previously reported ^11^. Then, the cells were stained with anti-CD4-FITC (BD Pharmingen), and subjected to FACS Canto (Becton, Dickinson and Company). The purity of the FACS-sorted CD4^+^ cell fraction as assessed by flow cytometry was >99%.

**Co-Culturing of Bone Marrow-Derived DCs and Splenic T-Cells**

The DCs and T-cells were resuspended in 24-well plates at a 1:10 ratio in serum-free RPMI 1640 medium with recombinant IL-2, recombinant IL-7, and recombinant TNF-α. Cells were pretreated for 20 h with 100 ng/ml LPS or PBS. Cells were recovered in standard medium for 24 h and were then incubated with 1 µg/ml OVA for 5 days at 37℃.

**Cytokine and Serum OVA-specific IgE Enzyme-Linked Immunosorbent Assays (ELISA)**

Cytokine concentrations (transforming growth factor (TGF)-β1, interferon (IFN)-γ, interleukin (IL)-4, IL-5, IL-13, IL-6, IL-2, IL-10, and IL-17A) in BALF and cell culture supernatants were measured using commercial ELISA kits according to the manufacturer's instructions (Xinbosheng, Shenzhen, China). To quantify OVA-specific IgE in serum samples, samples were analyzed using the murine OVA-specific IgE ELISA kit (Xinbosheng).

**Apoptosis Assay**

Cells were double-stained with propidium iodide (PI) and Annexin V-FITC according to the kit instructions (Boster Biological Technology, Wuhan, China). Flow cytometry was applied to determine the percentage of apoptotic cells, i.e., PI-negative/Annexin V-positive cells.

**Immunofluorescence**

As appropriate, cells were fluorescently labelled for CD11c (green anti-CD11c-FITC, 1:20, Becton Dickinson & Company, Mountain View, CA, USA), GITRL (red anti-GITRL, 1:200, GeneTex), and nuclei (blue, DAPI). Cells were fixed with formaldehyde and incubated with the primary antibody overnight at 4°C. Each experiment also used a negative control where PBS replaced the primary antibody. After thoroughly rinsing, a DyLight Cy3-conjugated secondary antibody was used at room temperature for 1 h. After rinsing, images were acquired at 60× magnification using a Nikon Eclipse E200 microscope connected to a Nikon Coolpix 995 camera (Nikon).

**Quantitative Reverse Transcription PCR (qRT-PCR)**

Total RNA was extracted with TRIzol reagent (Invitrogen, Carlsbad, CA, USA), and cDNA synthesis was performed using a PrimeScript RT Reagent Kit (Takara, Otsu, Japan). qRT-PCR) was performed using standard techniques. In each sample, β-actin was used as the internal control. The primer sequences are as follows: Foxp3, 5’-TGG AAC CAC GGG CAC TAT CAC A-3’ (forward) and 5’-GAG GCT GCG TAT GAT CAG TTA TGC-3’ (reverse); T-bet, 5’-AGC AAG GAC GGC GAA TGT T-3’ (forward) and 5’-GGG TGG ACA TAT AAG CGG TTC-3’ (reverse); GATA3, 5’-CTC GGC CAT TCG TAC ATG GAA-3’ (forward) and 5’-GGA TAC CTC TGC ACC GTA GC-3’ (reverse); RoR-γt, 5’-CCG CTG AGA GGG CTT CAC-3’ (forward) and 5’-TGC AGG AGT AGG CCA CAT TAC A-3’ (reverse); β-actin, 5’-GTC GCC CTG GAC TTC GAG C-3’ (forward) and 5’-GGT ACA TGG TGG TGC CGC CA-3’ (reverse).

**Cell Fractionation and Western Blotting**

Lung-derived CD11c^+^CD11b^+^ DCs isolated by flow cytometry were subjected to cellular fractionation and immunoblotting as previously described ^12^. Briefly, extraction of whole-cell protein content was accomplished using a lysis buffer (Beyotime, Jiangsu, China) containing 1 mM PMSF. Where indicated, extractions of nuclear and membrane proteins (as appropriate) were done with the Nuclear and Cytoplasmic Protein Extraction Kit (Beyotime) and Qproteome Plasma Membrane Protein Kit (Qiagen, Valencia, CA, USA), respectively. A BCA protein assay kit (Beyotime) was used to determine protein concentration. A total of 50 μg protein was then separated using 10% polyacrylamide gels before being electrotransferred to PVDF membranes from Millipore (Billerica, MA, USA). Using the manufacturer’s lowest dilution level, antibodies targeting GITRL (cat# GTX85076, GeneTex), cleaved caspase-3 (cat# 9661, Cell Signaling Technology, Danvers, MA, USA), TLR4 (cat# sc-293072, Santa Cruz Biotechnology), TRIF (cat# GTX13810, Genetex), phosphorylated and total TBK1 (cat# 5483 and 3013, Cell Signaling Technology), SARM (cat# 3295, ProSci, Poway, CA, USA), PTP1B and SIKE (cat# PA5-15521 and PA5-55247, Thermo Fisher Scientific, Waltham, MA, USA), SHP2 (cat# 3752, Cell Signaling Technology), and IFN-β (cat# ab85803, Abcam, Cambridge, UK) were immunoblotted onto the membrane. For the IRF3 immunoblots, antibodies against phosphorylated IRF3^Ser386^/phosphorylated IRF3^Ser386^ dimer (cat# PA5-37612, Thermo Fisher Scientific) and non-phosphorylated IRF3 monomer (cat# sc-376455, Santa Cruz Biotechnology) were employed using a previously described protocol ^13^. Endogenous β-actin provided the normalization control for whole-cell lysate blots, while Histone H3 and Na/K-ATPase provided the normalization controls for nuclear-fraction and membrane-fraction blots, respectively (cat# 3700, 14269, and 3010, respectively; Cell Signaling Technology). We employed the species-appropriate secondary IgG (H+L)-HRP antibodies (Bioworld). To quantify expression levels and normalize to control, the OD value of each band was calculated using Image J2x version 2.1.4.7 (Wayne Rasband, National Institutes of Health, Bethesda, MD, USA).

**Immunoprecipitation**

Cell extracts were pre-incubated with 20 µl protein G-agarose (50% slurry) at 4°C for 2 h. The beads were then removed by centrifugation, and the pre-cleared lysates were immediately incubated overnight with the appropriate antibodies (1-4 µg per sample) at 4°C. Immune complexes were then captured with protein G agarose (50 µl per sample) by incubation at 4°C for 4 h. The beads were then washed with ice-cold lysis buffer and resuspended in Laemmli buffer (Bio-Rad). Immunoprecipitates were then immunoblotted with the appropriate antibodies as described above.

**IRF3 Luciferase Reporter Assay**

Cells were cultured in Dulbecco's modified Eagle's medium (DMEM, Gibco, Shanghai, China) with 0.5 mg/ml neomycin (Sigma). In 24-well plates, 1×10^5^ cells/well were pretreated for 20 h with 100 ng/ml LPS or PBS, washed, and co-transfected with three plasmids with Superfect (Qiagen): a luciferase reporter containing the Gal4 upstream activation sequence (UAS-luciferase, 200 ng/well), Gal4-IRF3 (200 ng/well), and pRL-TK (Renilla luciferase, 50 ng per well). In control experiments, DBD-Gal4 was used in lieu of IRF3-Gal4. After LPS exposure and transfection, cells were recovered in standard medium for 24 h and were then incubated with 1 µg/ml OVA for 5 days at 37℃. The cells were then lysed with lysis buffer (Beyotime, Jiangsu, China), and the firefly and Renilla luciferase activities were assessed with a dual luciferase reporter assay system (Promega) on a luminometer (Suzhou SJ Biomaterials, Suzhou, China).

**Statistical Analyses**

Graph Pad Prism 7.0 (GraphPad, San Diego, CA, USA) and Excel 2013 (Microsoft, Redmond, WA, USA) were used for statistical analyses. Each experiment was repeated five times. Data are expressed as the means ± standard deviations (SDs). Two-way ANOVAs were used for LR and TLR4 KO analyses, while one-way analysis of variance (ANOVA) with Bonferroni post-hoc test or Student’s *t*-test were employed for other analysis. *P*-values of less than 0.05 were considered statistically significant.

1. Hirschfeld M, Ma Y, Weis JH, Vogel SN, Weis JJ. Cutting edge: repurification of lipopolysaccharide eliminates signaling through both human and murine toll-like receptor 2. *The Journal of Immunology.* 2000;165(2):618-622.

2. Goossens P, Gijbels MJ, Zernecke A, et al. Myeloid type I interferon signaling promotes atherosclerosis by stimulating macrophage recruitment to lesions. *Cell metabolism.* 2010;12(2):142-153.

3. Dewitz C, McEachern E, Shin S, et al. Hypoxia-inducible factor-1α inhibition modulates airway hyperresponsiveness and nitric oxide levels in a BALB/c mouse model of asthma. *Clinical Immunology.* 2017;176:94-99.

4. IKEDA M, KATOH S, SHIMIZU H, OKA M. Development of two types of mite-allergen induced murine models of chronic asthma with different severity. *Kawasaki medical journal.* 2016;42(1):1-7.

5. Bates JH, Irvin CG. Measuring lung function in mice: the phenotyping uncertainty principle. *Journal of Applied Physiology.* 2003;94(4):1297-1306.

6. van de Laar L, Guilliams M, Tavernier S. Isolation of conventional dendritic cells from mouse lungs. *Dendritic Cell Protocols.* 2016:139-152.

7. Xin H, Zhu J, Miao H, et al. Adenovirus-Mediated CCR7 and BTLA Overexpression Enhances Immune Tolerance and Migration in Immature Dendritic Cells. *BioMed research international.* 2017;2017.

8. Yu K-Y, Kim HS, Song SY, Min S-S, Jeong JJ, Youn B-S. Identification of a ligand for glucocorticoid-induced tumor necrosis factor receptor constitutively expressed in dendritic cells. *Biochemical and biophysical research communications.* 2003;310(2):433-438.

9. Wei SD, Li JZ, Liu ZJ, et al. Dexamethasone attenuates lipopolysaccharide‐induced liver injury by downregulating glucocorticoid‐induced tumor necrosis factor receptor ligand in Kupffer cells. *Hepatology Research.* 2011;41(10):989-999.

10. Kim HJ, Kim YJ, Lee SH, et al. Effects of Lactobacillus rhamnosus on asthma with an adoptive transfer of dendritic cells in mice. *Journal of applied microbiology.* 2013;115(3):872-879.

11. Lewkowicz N, Mycko M, Przygodzka P, et al. Induction of human IL-10-producing neutrophils by LPS-stimulated Treg cells and IL-10. *Mucosal immunology.* 2016;9(2):364-378.

12. Chen Z, Tang J, Cai X, et al. HBx mutations promote hepatoma cell migration through the Wnt/β‐catenin signaling pathway. *Cancer science.* 2016;107(10):1380-1389.

13. Robitaille AC, Mariani MK, Fortin A, Grandvaux N. A High Resolution Method to Monitor Phosphorylation-dependent Activation of IRF3. *Journal of Visualized Experiments Jove.* 2015;2016(107).
